# Genome-wide expression analysis reveals six contravened targets of EZH2 associated with breast cancer patient survival

**DOI:** 10.1038/s41598-019-39122-4

**Published:** 2019-02-13

**Authors:** Kanchan Kumari, Biswajit Das, Amit K. Adhya, Arabinda K. Rath, Sandip K. Mishra

**Affiliations:** 1 Cancer Biology Laboratory, Department of Cancer Biology, Institute of Life Sciences, Bhubaneswar, Odisha India; 2 Utkal University, Bhubaneswar, Odisha India; 3 Tumor Microenvironment and Animal Models Laboratory, Department of Translational Research, Institute of Life Sciences, Bhubaneswar, Odisha India; 4 Manipal University, Manipal, Karnataka India; 5 Department of Pathology, AIIMS, Bhubaneswar, Odisha India; 6 Hemalata Hospitals and Research Centre, Chandrashekharpur, Bhubaneswar, Odisha India

## Abstract

Several pioneering work have established that apart from genetic alterations, epigenetic modifications contribute significantly in tumor progression. Remarkable role of EZH2 in cancer highlights the importance of identifying its targets. Although much emphasis has been placed in recent years in designing drugs and inhibitors targeting EZH2, less effort has been given in exploring its existing targets that will help in understanding the oncogenic role of EZH2 in turn which may provide a more stringent method of targeting EZH2. In the present study, we validated six direct targets of EZH2 that are GPNMB, PMEPA1, CoL5A1, VGLL4, POMT2 and SUMF1 associated with cancer related pathways. Upon EZH2 knockdown, more than two fold increase in the target gene expression was evident. CHIP-qPCR performed in both MCF-7 and MDA-MDA-231 confirmed the *in-vivo* binding of EZH2 on its identified target. Thirty invasive breast carcinoma cases with their adjacent normal tissues were included in the study. Immunohistochemistry in primary breast tumor tissue array showed tumor dependent expression of EZH2. Array of MERAV expression database revealed the strength of association of EZH2 with its target genes. Real time PCR performed with RNA extracted from breast tumor tissues further authenticated the existing negative correlation between EZH2 and its target genes. Pearson correlation coefficient & statistical significance computed using the matrix provided in the database strengthened the negative correlation between identified target genes and EZH2. KM plotter analysis showed improved relapse-free survival with increased expression of PMEPA1, POMT2, VGLL4 and SUMF1 in breast cancer patients indicating their therapeutic potential. While investigating the relevance of these target genes, different mutations of them were found in breast cancer patients. Seeking the clinical relevance of our study, following our recent publication that reports the role of EZH2 in nicotine-mediated breast cancer development and progression, we observed significant reduced expression of SUMF1 in breast cancer patient samples with smoking history in comparison to never-smoked patient samples.

## Introduction

Understanding the basic genetic and epigenetic mechanisms underlying a disease is the key to identify new drug targets^1–3^. One of the globally recognized chromatin modifications is histone methylation that is reported to be associated with alterations in the gene expression contributing towards cancer. Histone methyltransferase activity of polycomb repressive group 2 (PRC2) is well studied in relation to cancer^4–9^. Enhancer of zeste homolog 2 (EZH2) is the catalytic subunit of PRC2 complex, expression of which is elevated in all cancers including breast cancer^10,11^. In recent years, numerous studies have been done in both human samples and animal models of cancer targeting EZH2^12,13^. Mutations and promising inhibitors have been developed to regulate its oncogenic function^14–18^. Genes related to cell cycle, epithelial to mesenchymal transition (EMT) pathways, DNA repair, apoptosis etc. have been recognized as EZH2 targets through several genome wide studies^12,19^. Both canonical and non-canonical role of EZH2 eventually insinuates towards the pleiotropy associated with this molecule, which is context dependent. Much attention is paid to understand the role of EZH2 in breast cancer and how it can be targeted. Systematic analysis of gene expression patterns using high throughput microarray analysis has led to the discovery of various genetic and epigenetic signatures in all cancers including breast cancer^20–24^, many of which remains to be cross validated. In addition, some studies have specifically studied the gene signature patterns extracted upon EZH2 knockdown or inhibition^25,26^. Biology of disease is equally important as fold change and p value for interpretation of microarray data. The acceptable value for statistically significant result often leads to small findings against a big question asked^3^. Answers to relevant questions that reside in the core of the disease such as breast cancer can be obtained from the critical analysis and interpretation of the data.

By analyzing publicly available CHIP-seq and gene expression datasets, we aimed at describing unexplored direct targets of EZH2 in breast cancer. Overall, in this study we validate six direct targets of EZH2 associated with patient survival, in breast cancer using published datasets and corroborate the existing co-relation between them in human primary breast carcinoma along with their adjacent normal tissues. Our data suggests the oncogenic role of EZH2 to be a consequence of coordinated action its target genes. In our recent publication, we have shown the enhanced expression of EZH2 playing significant role in nicotine-induced increased breast cancer progression. Correlating our previous report, the present study further signifies the finding by demonstrating the abrogated expression of SUMF1 in tissue sections from smoking breast cancer patients in comparison to never-smoked patient samples.

## Results

### Aberrant and tumor grade dependent EZH2 expression in breast carcinoma cells and primary breast tumors

**Figure 1:**
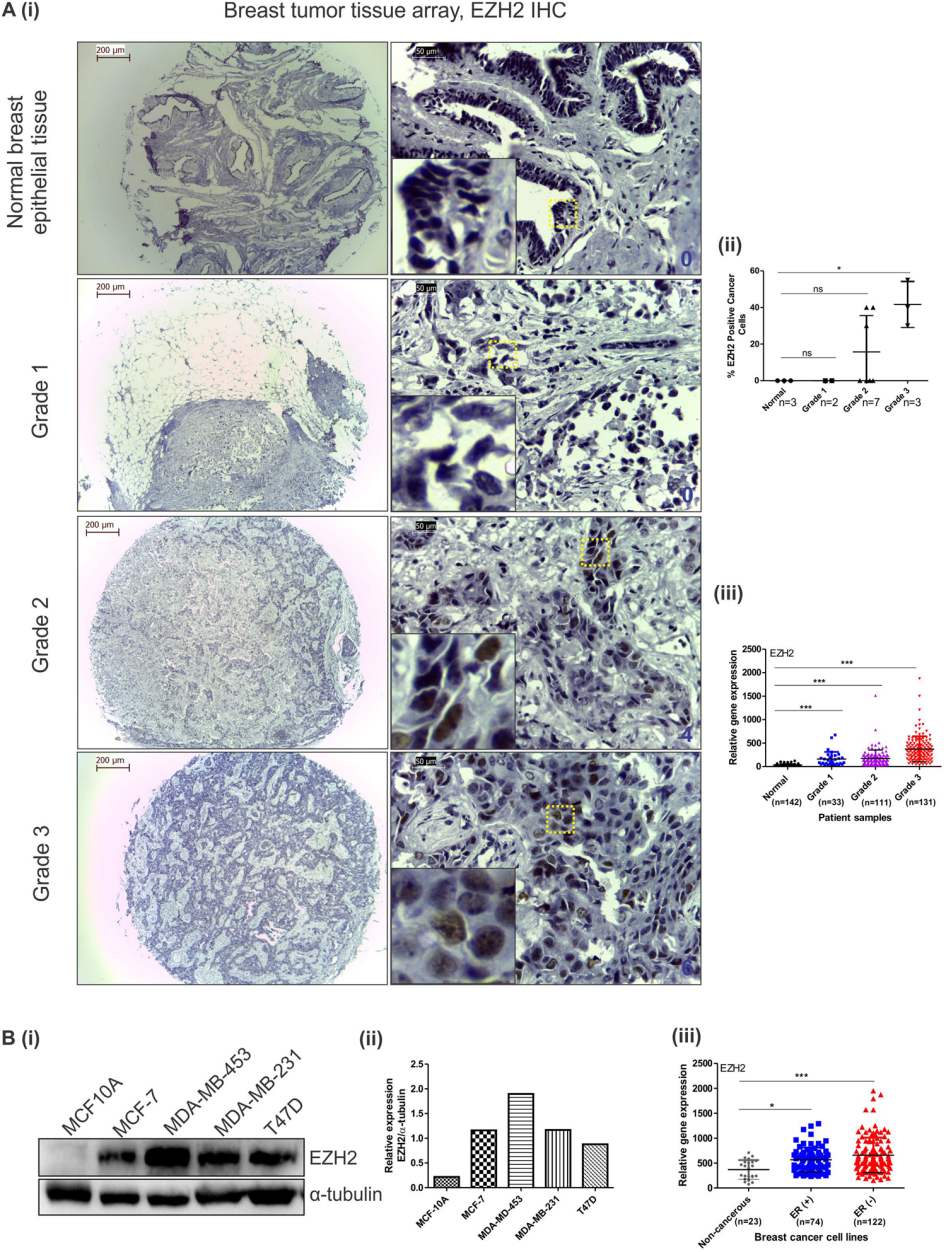
EZH2 expression increases with increasing tumor grade and is upregulated in breast cancer cell lines. (**A**) [i] Immunohistochemistry performed in tissue array of different grades of invasive breast tumors show EZH2 expression. Insets show 10 times digitally magnified pictures of images captured with 40X objective. [ii] The scatter plot shows the percent EZH2 positive cancer cells present in different grades of tumor tissues. [iii] Graph displays EZH2 expression in different grades of tumor in comparison to normal breast tissues as observed in MERAV expression database. (**B**) [i] Western blot displays EZH2 protein expression in breast cancer cell lines along with normal breast epithelial MCF10A cells. [ii] Graph shows normalized expression of EZH2 protein using ImageJ software. [iii] EZH2 expression in ER (+)ve and ER (−)ve breast cancer cells along with non-cancerous breast cells as observed in MERAV expression database is presented in the graph. One way ANOVA was used for statistical analysis. *P < 0.05, ***P < 0.001. Scale bar 200 µm (4X) and 50 µm (40X).

To explore and validate the previously defined role of EZH2 in breast cancer, we first investigated its expression in primary breast tissue array and different cell lines. In immunohistochemistry no expression of EZH2 was detected in normal breast tumors and grade 1 invasive ductal carcinoma tumors, whereas more than 2.5 fold increase in EZH2 expression was observed in grade 3 breast tumors when compared to grade 2 (Fig. 1Ai,ii). Although the sample size is small, the results authenticated EZH2 expression to be tumor grade dependent as previously predicted^6,27^. In MERAV expression database, there existed a significant difference in the expression of EZH2 in grade 1, grade 2 and grade 3 breast tumor when compared to normal breast tissue (Fig. 1Aiii). In comparison to normal breast epithelial cells, breast cancerous cells harbor enhanced EZH2 expression specifically in estrogen receptor (ER) negative breast cancerous cells in comparison to ER positive ones as detected by western blot assay (Fig. 1Bi,ii) thereby confirming the previous findings^28^. Similar pattern of EZH2 expression was observed in MERAV breast cancer cell line expression database (Fig. 1Biii).

### Estrogen receptor positive and negative breast carcinoma cells share common EZH2 targets

**Figure 2:**
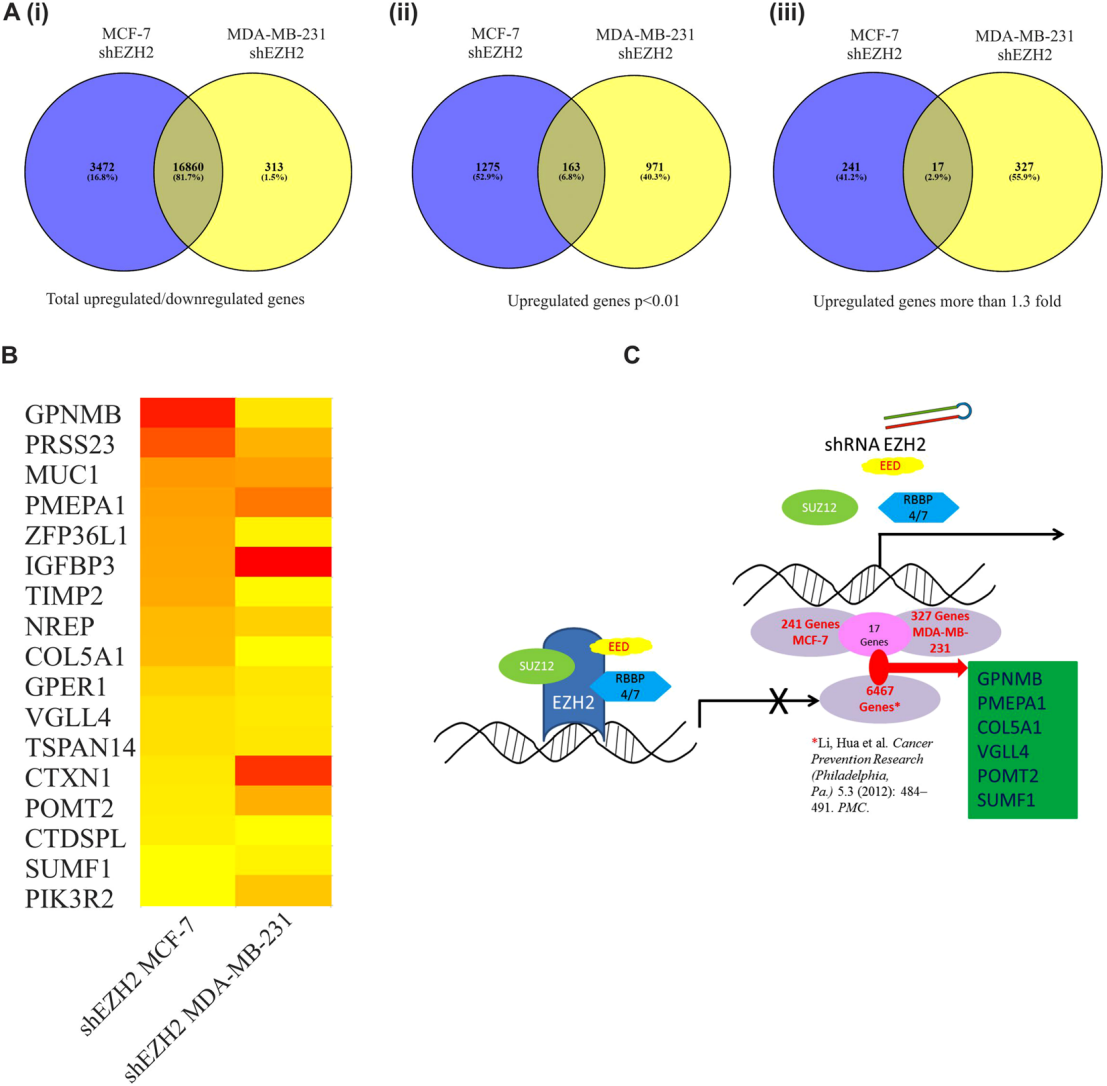
Identification of unexplored direct EZH2 target genes commonly upregulated in EZH2 depleted MCF-7 and MDA-MB-231 breast cancer cell lines. (**A**) Venn diagrams depict the number of genes in EZH2shRNA transfected MCF-7 and MDA-MB-231 breast cancer cells lines analyzed from published GEO datasets. [i] Figure shows the percent of overlapping genes in the two different cell types. [ii] Number of differentially upregulated genes common in both cell types with p value less than 0.01 is shown in the diagram. [iii] Diagram shows common genes upregulated by more than 1.3 fold. (**B**) Heat map shows the expression of the 17 differentially upregulated genes in both the cell types. (**C**) Diagram displays the identification of six direct targets of EZH2 using gene expression profiling and CHIP-seq datasets. Software Venny 2.1.0 was used to create Venn diagrams.

It is well established that steroid hormones play crucial role in breast transformation and that the presence of hormone receptor such as ER alpha allows the patients to response to hormone therapy. Hormone unresponsive breast cancer called triple negative breast cancer is more aggressive and metastatic^29^. Therefore, the disease seeks research that identifies drug targets common to both the cancer types. To find EZH2 target genes in breast cancer common to both ER positive and ER negative breast cancer we chose MCF-7 and MDA-MB-231 gene expression profiling arrays performed upon EZH2 knockdown. Eighty-two percent genes (16860) were commonly altered upon EZH2 knockdown in both MCF-7 and MDA-MB-231 cell lines (Fig. 2Ai). Among them, 7 percent of the genes (p < 0.01) (163) were commonly upregulated (Fig. 2Aii). Seventeen genes were found to be upregulated by more than 1.3 fold upon EZH2 knockdown in both the cell types (Fig. 2B). EZH2 globally occupied six out of seventeen commonly upregulated genes as observed in the CHIP-seq data set (Fig. 2C). Thus, we selected six unexplored direct EZH2 targets namely Glycoprotein Nonmetastatic Melanoma Protein B (GPNMB), Prostate Transmembrane Protein Androgen Induced 1 (PMEPA1), Collagen Type V Alpha 1 Chain (CoL5A1), Transcription Cofactor Vestigial-Like Protein 4 (VGLL4), Protein O-Mannosyltransferase 2 (POMT2) and Sulfatase Modifying Factor 1 (SUMF1) for further validation.

### Reduced or increased EZH2 expression alters the expression of its identified target genes

**Figure 3:**
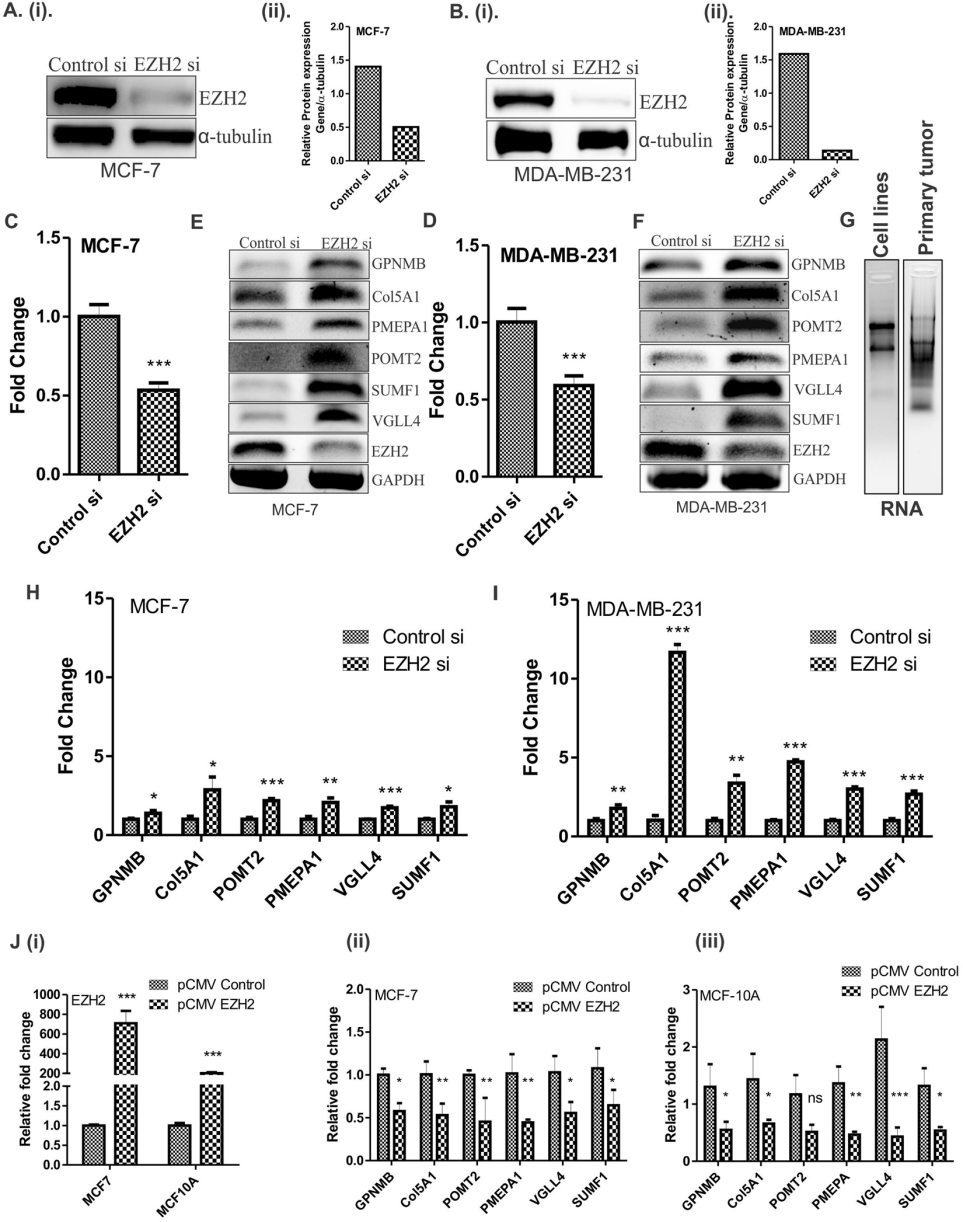
Expression of identified target genes corresponds to EZH2 expression in breast cancer cells. (**A**) [i] and (**B**) [i] Immunoblot depicts the level of EZH2 knockdown in MCF-7 and MDA-MB-231 cells respectively. (**A**) [ii] and (**B**) [ii] Graphs display respective ImageJ quantification of EZH2 protein in MCF-7 and MDA-MB-231 upon EZH2si transfection. (**C**,**D**) Relative fold change of EZH2 expression in controlsi and EZH2si transfected MCF-7 and MDA-MB-231 cells respectively is displayed in the graphs. (**E**,**F**) Agarose gel picture shows the expression of EZH2 and its target genes in MCF-7 and MDA-MB-231 respectively upon EZH2 knockdown using semi-quantitative PCR. (**G**) Figure shows the representative image for quality of RNA isolated from breast cancer cell lines and primary breast tumors. (**H**,**I**) qRT-PCR data displayed in the graph shows real time expression of identified target genes upon EZH2 knockdown in MCF-7 and MDA-MB-231 respectively. (**J**) [i] Relative fold change in EZH2 expression upon transfection of its over-expression construct is evident from the graph. [ii] and [iii] Graph depicts the mRNA expression of EZH2 target genes upon ectopic expression of EZH2 in MCF-7 and MCF-10A respectively. Two tailed paired Student t-test and one way ANOVA was used for statistical analysis for experiments done in triplicate *P < 0.05, **P < 0.005, ***P < 0.001.

To study the effect of EZH2 expression on its target genes, we checked their expression upon EZH2 knockdown and over-expression. Target genes were significantly up-regulated in both MCF-7 and MDA-MB-231 breast carcinoma cell lines upon EZH2 knockdown. Fifty percent knockdown in the level of EZH2 in MCF-7 (Fig. 3A,C) and MDA-MB-231 (Fig. 3B,D) resulted in significant increase in the target gene expression (Fig. 3E,F) as observed at transcript level. Upon EZH2 knockdown, fold change in the target gene expression for GPNMB, CoL5A1, POMT2, PMEPA1, VGLL4 and SUMF1 was found to be 1.3, 2.8, 2.2, 2, 1.7 and 1.8, respectively in MCF-7 breast carcinoma cells as detected by qPCR (Fig. 3H). At the same time, in aggressive breast cancer cells MDA-MB-231, the respective fold change in the expression of target genes was 1.8, 11.7, 3.4, 4.7, 3 and 2.7 (Fig. 3I). Further to check the expression of identified target genes in an EZH2 overexpressed state, we transfected pCMV-EZH2 (Addgene) in breast cancer cells (Fig. 3Ji). Ectopic expression of EZH2 in MCF-7 cells resulted into 42, 47, 54, 48, 46 and 40 percent reduced expression of GPNMB, CoL5A1, POMT2, PMEPA1, VGLL4 and SUMF1 respectively (Fig. 3Jii). As MCF-7 cells have moderate level of EZH2, we also performed qRT-PCR assay in normal breast epithelial cells MCF10A, that harbor very low level of EZH2 expression. Similar to the results obtained in MCF-7 cells, more than 50 percent reduction in all the target gene expression (Fig. 3Jiii) was observed in EZH2 over-expressed MCF10A cells.

### EZH2 regulates its identified target genes by direct binding

**Figure 4:**
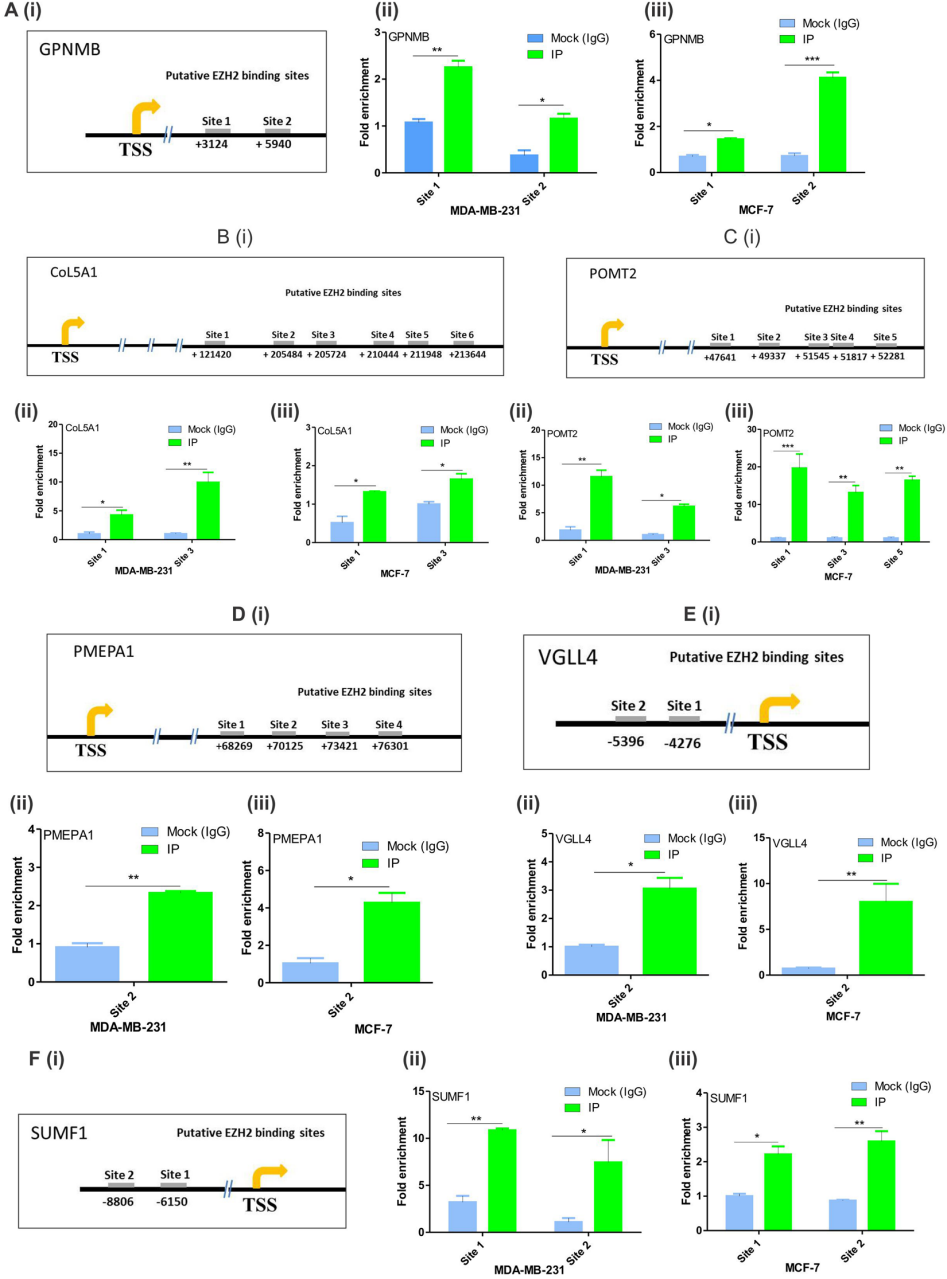
*In-vivo* binding of EZH2 on its target genes. (**A**) [i], (**B**) [i], (**C**) [i], (**D**) [i], (**E**) [i], and (**F**) [i] Diagrams show putative EZH2 binding sites of GPNMB, CoL5A1, POMT2, PMEPA1, VGLL4 and SUMF1 from the transcription start site (TSS). (**A**) [ii], (**B**) [ii], (**C**) [ii], (**D**) [ii], (**E**) [ii] and (**E**) [ii], Graphs display the fold enrichment of EZH2 on the putative binding sites of GPNMB, CoL5A1, POMT2, PMEPA1, VGLL4 and SUMF1 respectively in MDA-MB-231 cells. (**A**) [iii], (**B**) [iii], (**C**) [iii], (**D**) [iii], (**E**) [iii], and (**F**) [iii] Graphs display the fold enrichment of EZH2 on the putative binding sites of GPNMB, CoL5A1, POMT2, PMEPA1, VGLL4 and SUMF1 respectively in MCF-7 cells. Two tailed paired Student t-test and one way ANOVA was used for statistical analysis for experiments done in triplicate *P < 0.05, **P < 0.005, ***P < 0.001.

To further confirm the direct gene-target association between EZH2 and its identified targets, we performed CHIP-qPCR following *in-vivo* binding assay CHIP with EZH2 antibody. CHIP-seq dataset analyzed in the study report several putative EZH2 binding sites on its target genes. Both the two upstream putative EZH2 binding sites on GPNMB were found to be occupied by EZH2 (Fig. 4A) in both the cell lines. Out of six putative downstream EZH2 binding sites on CoL5A1, only site 1 and site 3 were bound by EZH2 in the cells (Fig. 4B). Similarly in case of POMT2, EZH2 was found to occupy site 1 and site 3 in MDA-MB-231 cells and site 1, 3 and 5 in MCF-7 cells (Fig. 4C). Only one binding site (site 2) of both PMEPA1 (Fig. 4D) and VGLL4 (Fig. 4E) were found to be positive for EZH2 out of four and two putative sites respectively in both the cell lines. EZH2 was found to bind to both the two putative upstream binding sites of SUMF1 (Fig. 4F).

### Strength of association between EZH2 and its target genes

**Figure 5:**
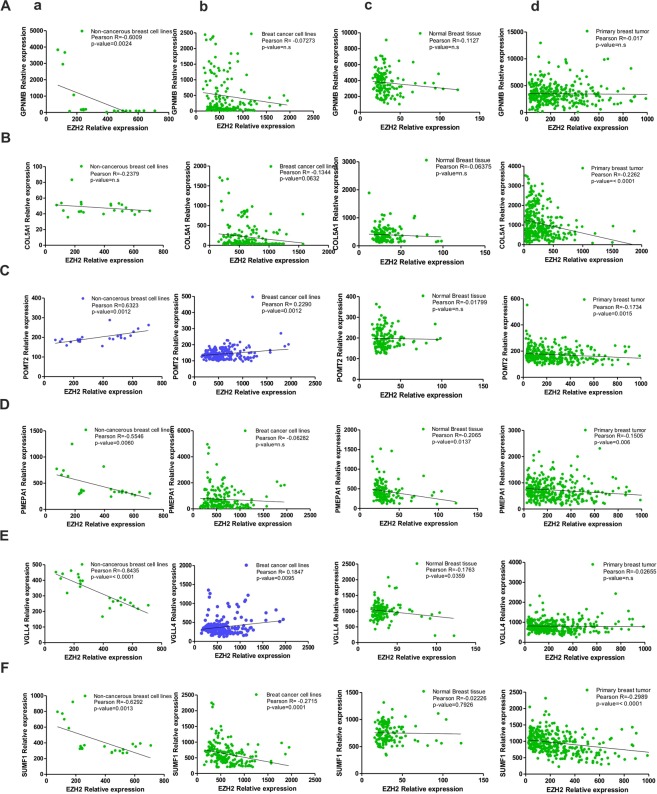
EZH2 and target gene correlation as observed in MERAV database. (**A**–**F**) Graphs show respective correlation of expression of GPNMB, COL5A1, POMT2, PMEPA1, VGLL4 and SUMF1 with EZH2 expression in non-cancerous breast cell lines (a), breast cancerous cell lines (b), normal breast tissues (c) and in primary breast tumors (d). Pearson correlation coefficient between the variables was calculated with the help of the software GraphPad Prism v5.01.

Correlation coefficients are used to study the strength of association between two genes^30^. A correlation coefficient of zero indicates that no linear relationship exists between two genes, and a correlation coefficient of −1 or +1 indicates a perfect linear relationship. The strength of relationship can be anywhere between −1 and +1 showing the strength of the putative linear association between them; a positive number indicates positive correlation and a negative number indicates inverse correlation between them. If EZH2 regulates the expression of its identified targets by its methyltransferase activity, a negative value of Pearson correlation coefficient is expected. Therefore, to define the type of association between EZH2 and its targets, Pearson correlation coefficient values were calculated using the partial matrix values provided in the MERAV database. In non-cancerous cell line expression dataset, strong negative correlation (coefficient values r = −0.8435 for VGLL4 and r = −0.2379 for CoL5A1) was found between EZH2 and its target genes except for POMT2 where a significant positive relation was evidenced (r = 0.6323) (Fig. 5a). A weak negative correlation was observed between EZH2 and its targets in breast cancer cell lines MERAV dataset (except for POMT2 and VGLL4 where a positive correlation was detected) (Fig. 5b). In normal (Fig. 5c) as well as primary breast tissues (Fig. 5d), negative correlation coefficient values were witnessed between EZH2 and its targets.

### Both non-cancerous and cancerous breast cells harbor negatively correlated expression of EZH2 and its identified targets

**Figure 6:**
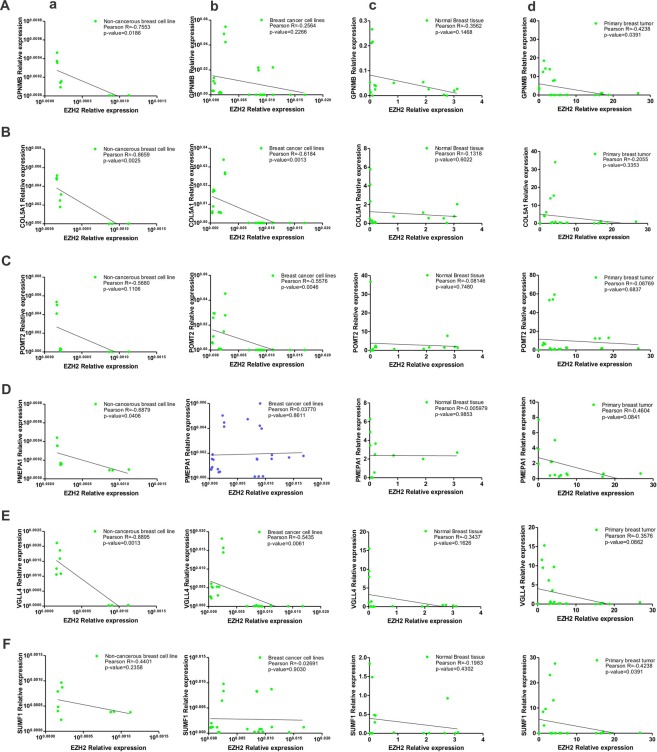
Correlation of expression of EZH2 and its identified targets in breast tumor samples and breast cell lines as obtained from quantitative real time PCR following RNA extraction. (**A**–**F**) Graphs show the existing correlation of EZH2 expression and its target gene expression in non-cancerous breast cell lines (a), breast cancerous cell lines (b), normal breast tissues (c) and in primary breast tumors (d) respectively as computed by qRT-PCR data. Pearson correlation coefficient between the variables was calculated with the help of the software GraphPad Prism v5.01.

To corroborate the expression data of MERAV database, were checked the expression status of EZH2 and its target genes by quantitative real time PCR in different breast cancer cell lines. In MCF10A normal breast epithelial cells, similar to MERAV expression database of non-cancerous breast cell lines, a strong negative correlation highest for VGLL4 (r = −0.8895) and lowest for SUMF1 (r = −0.4401) was observed between EZH2 and its target genes (Fig. 6a). In qRT-PCR data obtained from MCF-7, T47D, MDA-MB-231 and MDA-MB-453 breast cancer cell lines, a negative correlation was observed between EZH2 and its target gene expression except for PMEPA1 that shared a weak positive correlation with EZH2 (Fig. 6b).

### A negative correlation exists between EZH2 and its targets in histologically similar primary breast tumors and adjacent normal breast tissues

Further to correlate the MERAV breast tumor expression database, we studied the correlation in the expression of EZH2 and its target genes by quantitative real time PCR following RNA extraction from both tumor and adjacent normal tissues collected from histologically similar primary breast tumors. EZH2 negatively correlated its target genes in adjacent normal breast tissues of breast cancer patient included in the study (Fig. 6c). In primary breast tumor tissues a significant negative correlation was evidenced between EZH2 and its target genes (Fig. 6d) which corroborated the correlation observed in online MERAV database.

### Identified unexplored targets of EZH2 are associated with relapse free survival in breast cancer patients

**Figure 7:**
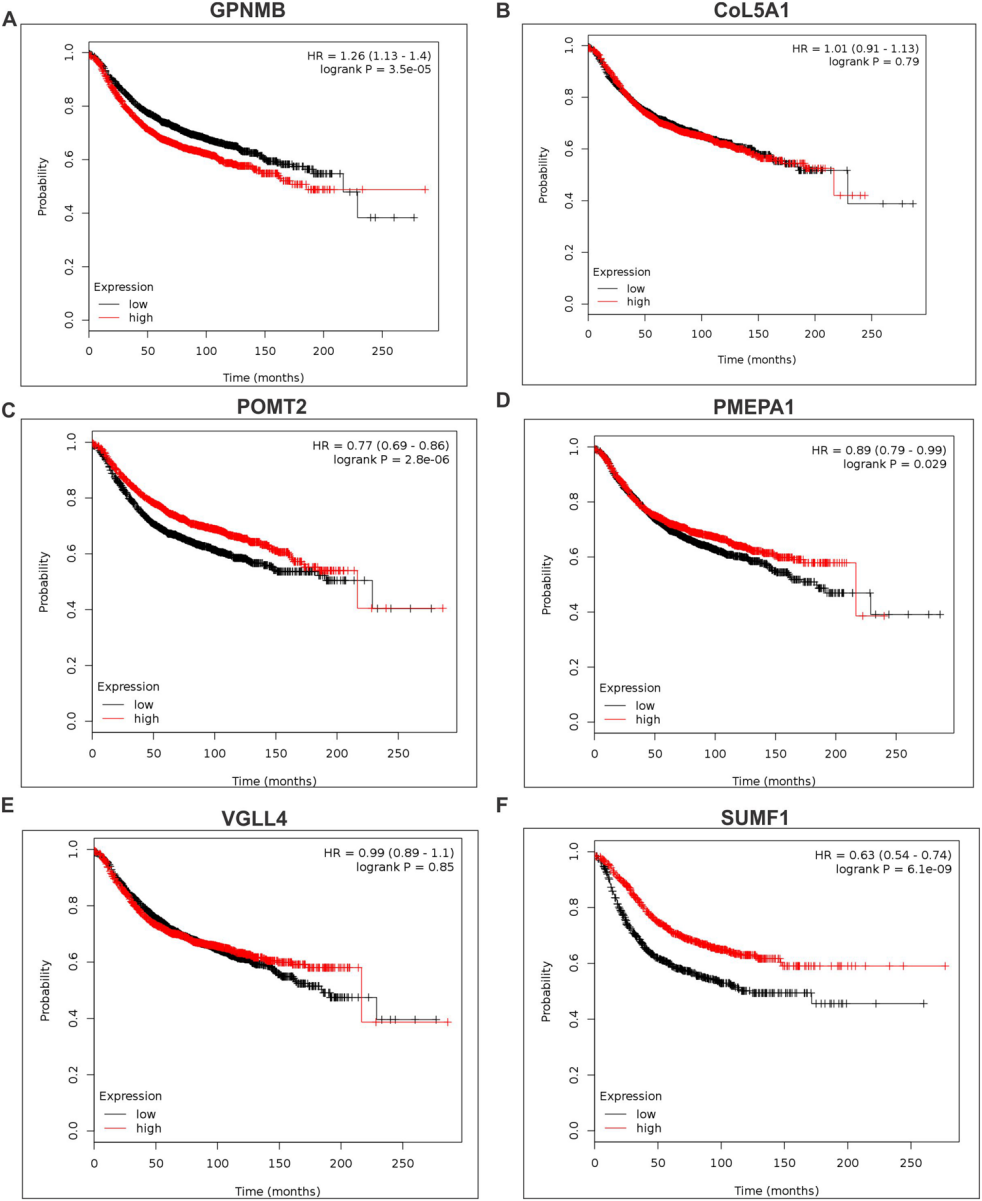
Identified target genes are associated with breast cancer patient survival. (**A**–**F**) Kaplan Meier plots show the survival curves of GPNMB, CoL5A1, POMT2, PMEPA1, VGLL4 and SUMF1 in breast cancer patients. Kaplan-Meier survival curves plot fractional survival (Y) as a function of time (X).

High expression of GPNMB (Fig. 7A) is associated with reduced relapse free survival indicating its oncogenic possessions. A concomitant improved relapse free survival in breast cancer patients with increased expression of POMT2 (Fig. 7C), PMEPA1 (Fig. 7D) and SUMF1 (Fig. 7F) indicated their tumor suppressive behavior. Association of CoL5A1 (Fig. 7B) and VGLL4 (Fig. 7E) expression with patient survival is not very conclusive as the log rank p-value is more than 0.05.

### Association of identified EZH2 targets with aggressiveness of the disease

Increasing tumor grade and estrogen receptor dependent expression of any gene indicates its association with aggressiveness of the disease. Therefore, to evaluate the association of identified EZH2 target genes with breast cancer, we investigated their expression in various cancerous cell lines including non-cancerous as well as primary breast tumors and normal breast tissues in MERAV expression database.

**Figure 8:**
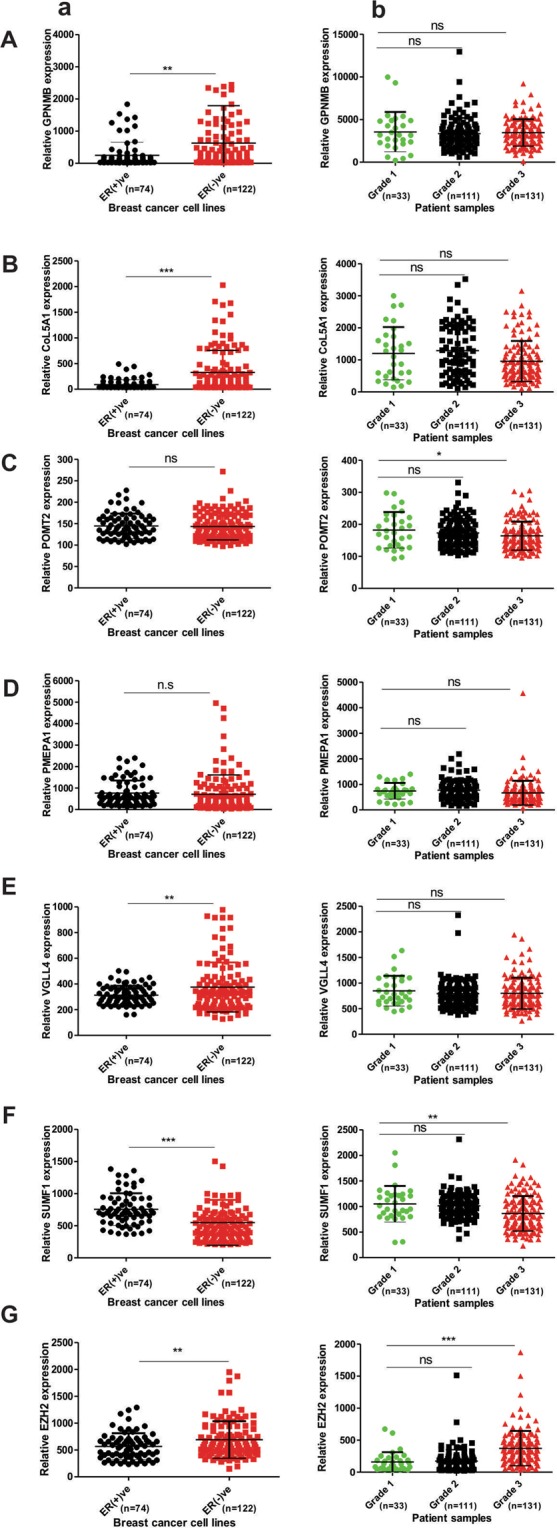
Estrogen receptor and tumor grade dependent expression of identified EZH2 target was studied. (**A**–**G**) Respective expression of GPNMB, COL5A1, POMT2, PMEPA1, VGLL4, SUMF1, and EZH2 in ER (+)ve and ER (−)ve breast cancer cells (a), and different breast tumor grades (b) of online MERAV dataset. Two tailed paired Student t-test was used for statistical analysis *P < 0.05, **P < 0.005, ***P < 0.001.

Correlating the survival curve analyses, a respective 2.5 and 3.7 fold enhanced expression of GPNMB and CoL5A1 was evidenced in ER negative breast cancer lines in comparison to ER positive ones in MERAV expression database (Fig. 8Aa,Ba). However, their expressions were found to be tumor grade independent (Fig. 8Ab,Bb). ER independent expression of POMT2 was observed in breast cancer cells (Fig. 8Ca). However, its expression in grade 3 tumor samples was found to be significantly reduced when compared to grade 1 tumors (Fig. 8Cb). PMEPA1 expression was found to be independent of tumor grade and estrogen receptor status (Fig. 8D). Tumor grade independent but a significant difference in expression of VGLL4 was detected in ER positive and negative breast cancer cells (Fig. 8E). Corroborating the survival curve data, SUMF1 expression in more aggressive ER negative breast cells and higher-grade tumor was found to be abrogated (Fig. 8F). EZH2 expression already compared with normal breast tissues and non-cancerous breast cells (Fig. 1Aiii,Biii) is enhanced in ER negative breast cells in comparison to ER positive breast cancerous cells (Fig. 8G,a). Also when compared with grade 1 tumors, expression of EZH2 in grade 2 tumor was unaffected but was found to be significantly increased in grade 3 tumors (Fig. 8G,b) corroborating the previously reported data and IHC data presented in Fig. 1.

### Mutations of EZH2 targets in breast cancer

**Figure 9:**
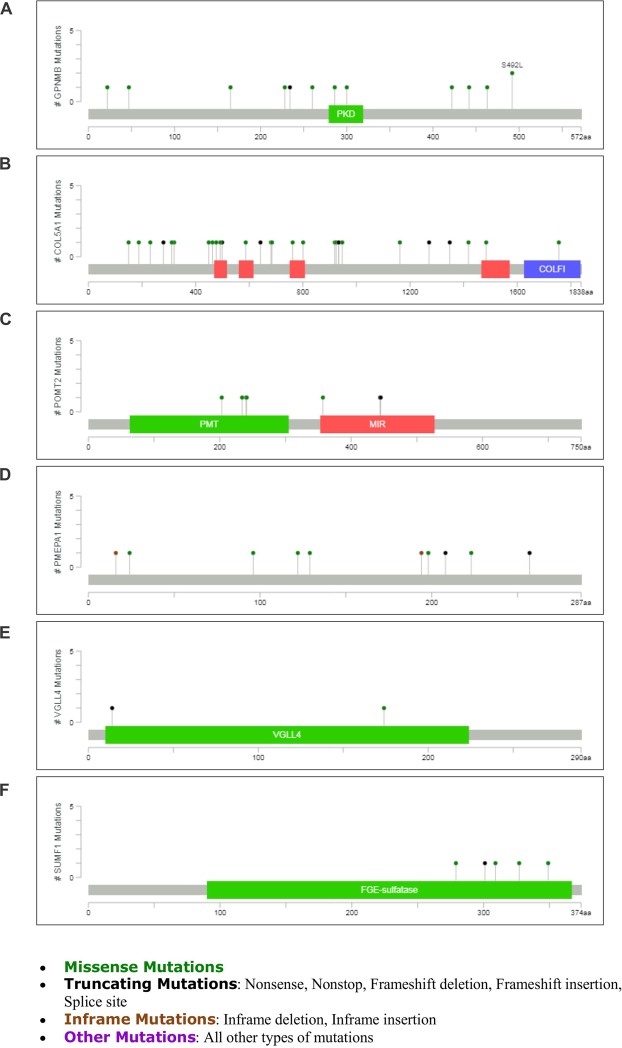
Mutations found in identified EZH2 target genes. (**A**–**F**) Lollipop graphs show the type and number of mutations of GPNMB, COL5A1, POMT2, PMEPA1, VGLL4, SUMF1, respectively in breast cancer. The graphs were downloaded from cBioPortal, a web source for cancer genomics. Mutation diagram circles are colored differently for different mutation types. In case of multiple mutation types at a single position, circle is colored by the most frequent mutation observed at that particular position.

To further study the association of identified EZH2 target genes with breast cancer, we explored the type/number of mutations observed in them in online cancer genomics data. 23 Mutations (includes 9 duplicate mutations) for GPNMB (Fig. 9A) was found that included various missense mutations such as amino acid changes A22T, P260L, S442R, N300D, S492L etc. GPNMB contains PKD (Polycystic Kidney Disease) domain identified in PKD protein polycystin-1 having role in protein–protein and protein–carbohydrate adhesive interactions. Highest number of mutations (53, including 24 duplicate mutations) was found for CoL5A1 (Fig. 9B) that included the missense mutations in Laminin G domain, Collagen triple helix repeat sites and Fibrillar collagen C-terminal domain of the gene. In POMT2 19 mutations (includes 11 duplicate mutations) were found majorly in Dolichyl-phosphate-mannose-protein Mannosyltransferase (PMT) domain and MIR [named after proteins in which it occurs: protein Mannosyltransferase, Inositol 1,4,5-trisphosphate receptor (IP3R) and Ryanodine receptor (RyR)] domain (Fig. 9C). Frame-shift, in-frame, missense and non-sense deletions were found in 22 different type of mutations (includes 6 duplicate mutations) for PMEPA1 (Fig. 9D) in breast cancer. Five specific mutations were seen for VGLL4 (Fig. 9E) and 9 mutations (includes 2 duplicate mutations) for SUMF1 (Fig. 9F) was observed. The protein product of the SUMF1 gene is FGE-sulfatase, formylglycine (FGly),-generating enzyme that play important role in degradation and remodeling of sulfate esters^31^.

### EZH2 functions through its target gene

**Figure 10:**
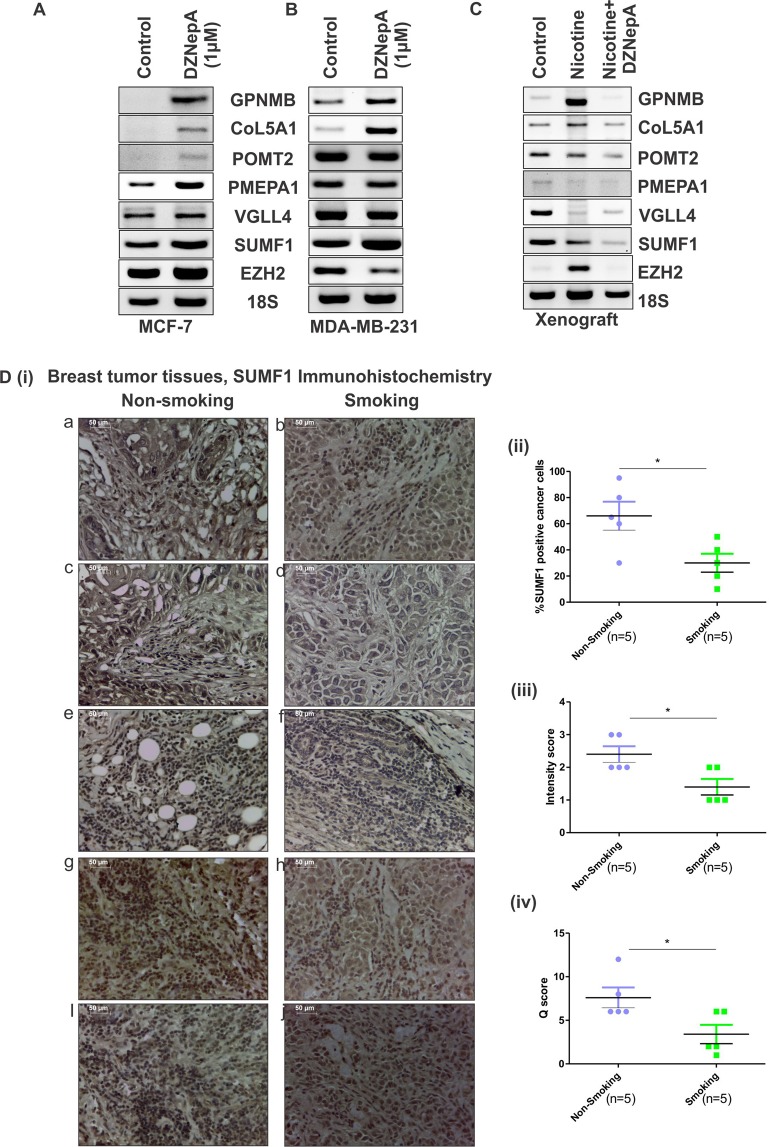
EZH2 functions through its target genes. (**A**,**B**) Diagram shows the mRNA expression of EZH2 and its target genes in control and DZNepA treated MCF-7 and MDA-MB-231 breast cancer cells respectively observed in reverse transcriptase PCR. (**C**) Reverse transcriptase PCR data shows expression of EZH2 and its target genes in xenograft collected from vehicle, nicotine and nicotine & DZNepA treated mice. (**D**) [i] Expression of SUMF1 in tissue sections from smoking and non-smoking breast cancer patients as observed in immunohistochemistry. [ii] Scatter plot depicts the percent SUMF1 positive cancer cells in the tissue section from smoking and non-smoking patient samples. [iii] Scatter plot shows the intensity score of SUMF1 expression in two experimental groups. [iv] Graph displays the Q-score of SUMF1 expression in experimental groups. Two tailed paired Student t-test was used for statistical analysis *P < 0.05.

To investigate the clinical relevance of our study, we explored the association of EZH2 in real scenario. In our recent study^32^, we report significant role of EZH2 in nicotine-induced increased breast cancer progression. An enhanced expression of EZH2 was observed in nicotine treated breast cancer cells, xenografts and breast cancer patients with smoking history in comparison to non-smoking samples. Using EZH2 inhibitor DZNepA we validated the involvement of methyltransferases such as EZH2 in nicotine associated tumorigenesis. As DZNepA treatment reduces EZH2 protein by increased degradation^4^, we went ahead to check the expression of identified EZH2 targets upon DZNepA treatment in MCF-7 and MDA-MB-231 by reverse transcriptase PCR. Although the mRNA expression of EZH2 was not affected upon DZNepA treatment in MCF-7, the expression of its target genes were found to be up-regulated except for VGLL4 expression that remain unaltered (Fig. 10A). In MDA-MB-231 breast cancer cells, reduced mRNA expression of EZH2 was detected upon DZNepA treatment whereas increased expression of its target genes (except POMT2 and VGLL4) was found (Fig. 10B). To investigate the expression status of identified EZH2 target genes in nicotine treated condition, we also checked their expression in RNA extracted from xenograft of vehicle, nicotine, and nicotine & DZNepA co-treated nude mice. Interestingly, increased EZH2 expression harboring nicotine treated samples showed reduced expression of its target genes in comparison to vehicle treated samples (Fig. 10C). Expression trend of CoL5A1 and GPNMB was similar to EZH2 expression in nicotine treated samples correlating the previously observed KM plotter and correlation data suggesting their oncogenic behavior. Further, to validate the correlated expression of EZH2 targets and EZH2 in conditions such as nicotine consumption, we performed immunohistochemistry with SUMF1, the target showing most significant association with breast cancer patient survival, in tissue sections of smoking and non-smoking breast tumors. Five pairs of appropriately matched^32^ smoking and non-smoking breast cancer samples were stained with SUMF1 antibody (Fig. 10D,i). Correlating the mRNA expression data, a significantly reduced SUMF1 positive cancer cells was observed in nicotine exposed breast cancer patients when compared to never-smoked patient samples (Fig. 10D,ii).

## Discussion

Key roles of EZH2 in cancer progression rationalize the investigation of its existing targets. Acceptable fold change cut-off values and significance of particular p-values lead to consideration of very few numbers of genes to be evaluated in microarray genes^33^. So there is a debate for whether biology or the statistical cut-offs is more important in interpretation of the data which often leads to narrow statements out of a big scientific question^3^. Here in this study by taking gene overexpressed by more than 1.3 fold as cut-off we gave importance to the biology of cancer to identify the unexplored existing targets of global gene repressor EZH2. Our semi-quantitative and quantitative real time data validated six direct targets of EZH2 significantly associated with the breast cancer. CHIP-qPCR further confirmed EZH2 binding on its target genes. Examination of correlation between EZH2 and its identified target genes in breast tissues both cancerous and non-cancerous revealed a negative correlation between EZH2 and its targets with some deviation. Interestingly, POMT2 positively correlated with EZH2 in breast cancer cells along with non-cancerous cell lines whereas in primary breast tumor and normal breast tissues, a negative correlation existed between the two. At the same time, in MERAV expression databaase, EZH2 and VGLL4 showed a negative correlation in non-cancerous breast cell lines and normal breast tissues, which reversed in cancerous cell lines. In breast tumor samples, the existing association between EZH2 and its target genes correlated the findings from gene expression MERAV database with some variation. A negative correlation existed between EZH2 and its targets except in PMEPA1, which showed a weak positive correlation in breast cancer cell lines.

Inter and intra-tumoral heterogeneity underlies the diverse pattern of correlation significance between EZH2 and the genes targeted by it. The MERAV expression database consists of different histological types of tumor such as Invasive Ductal, Ductal, Papillary, Medullary, Lobular, Inflammatory, Mucinous, Metaplastic Squamous Carcinoma etc. Also, varied strength of correlation was observed between breast non-cancerous/cancerous cell lines and normal/cancerous tissues, which can be, explained from the fact that cell lines are derived from tumors and are grown and expanded in culture *in vitro*. Cell lines acquire changes in the process of immortalization and during maintenance in culture. Several studies in different cancers have explored and compared the genomic profiles of cell lines and cancerous tissues^34–36^.

The real breast tumor samples used in the study is from same histological type of tumor that is invasive breast carcinoma. In the data obtained by quantitative real time following RNA extraction from tissues samples showed significant negative correlation between EZH2 and the target genes. While investigating the association of the identified EZH2 target genes, we found two of them are good for cancer as oncogenes and the function of other four suggests them to serve as tumor suppressor genes. Physiological and potential pathological role of GPNMB is reported in both normal and cancerous tissue. Elevated expression of GPNMB in all cancer and its remarkable role in breast tumor progression^37–42^ has proven it to be a potential therapeutic target. Association of CoL5A1 with EMT and thus with cancer metastasis is described in muscle invasive bladder transition cell carcinoma and glioblastoma^43^. Its involvement in collagen remodeling in serous ovarian cancer is reported^44^. POMTs play crucial role in embryogenesis and development^45,46^. Studies demonstrate the crucial role of O-mannosylation in cell-cell adhesion. O-mannosyl glycans alter E-cadherin expression that is the most essential protein involved in cell-cell adhesion^45^.

PMEPA1 functions as a negative regulator of TGFβ signaling and thus plays important role in tumorigenesis and bone metastasis in prostate cancer^47,48^. Although survival curve showed significant association of increased expression of PMEPA1 with improved survival in breast cancer, studies report its upregulated expression in breast cancer and its role in promoting cancer stem cell population^49^, which warrants further research.

VGLL4 known for its tumor suppressive role in pancreatic cancer^50^ competes with yes associated protein for binding to transcriptional enhancer factor (TEAD) and inhibits YAP induced tumorigenesis^51,52^. Another putative tumor suppressor gene identified as EZH2 target is SUMF1. SUMF1 plays important role in the activation of sulfatases involved in various processes such as remodeling of heparin sulfate proteoglycans that have shown to affect hematopoietic stem cell self-renewal and differentiation^53^. Relation of sulfatases and Sulfatase Modifying Factors are well studied^54^. Mutations in SUMF1 gene has led to many disorders such as lysosomal storage disorders^55,56^. Regulation of SUMF1 by miR-95 is involved in cellular proliferation has shed light on its possible role in carcinogenesis. Also effect of sulfation and desulfation in steroid action encourages the idea of its involvement in tumorigenesis^57^.

Abrogated expression of SUMF1 in smoking associated patient samples where our group has previously showed enhanced EZH2 expression strengthens the association and correlation between EZH2 and its target gene. In addition, our study dictates that EZH2 may regulate both oncogenes and tumor suppressor genes. GPNMB and CoL5A1 are the reported oncogenes; PMEPA1, POMT2, VGLL4 and SUMF1 show better survival and thus suggest tumor suppressive role are being regulated by EZH2 signifying its dual role. Regulation of GPNMB and CoL5A1 by EZH2 endorses its two faces in cancer^7^. Overexpressed EZH2 mediated repression of these genes reveals its multifaceted role in cancer. EZH2 plays crucial role in breast cancer progression through its target genes as identified in this study, thereby signifying that EZH2 target genes may provide a more stringent method of targeting EZH2. Although functions of identified EZH2 target genes are known, their role and regulation in cancer is not well studied and may prove to be a therapeutic target in breast cancer treatment.

## Materials and Methods

### Ethics statement

All breast cancer specimens were collected during operation with written informed consents from the patients and were approved by Institutional Human Ethical Committee (Institute of Life Sciences, Bhubaneswar, India). All human tumor samples were handled in accordance with an approved protocol of human ethical committee.

### Data sets

To identify the unexplored EZH2 targets in breast cancer, we analyzed the publically available gene expression microarray datasets in spite of mere repetition of the profiling. Gene expression microarray datasets for EZH2 knock down in MDA-MB-231^58^ and MCF-7^59^ cell lines was obtained from Gene Expression Omnibus (GEO Accession Number GSE30670 and GSE68050). Both datasets were conducted on same platform Illumina. These datasets were used to identify the targets of EZH2 common to both estrogen receptor positive MCF-7 and estrogen receptor negative MDA-MB-231 breast carcinoma cells. Using the list of genes differentially expressed in both the datasets upon EZH2 knockdown, we checked for commonly upregulated genes (p < 0.01) more than 1.3 fold. To validate the global occupancy of EZH2 on its identified target genes, the CHIP-seq data generated by Li *et al*.^60^ was analyzed.

### Chromatin immunoprecipitation assay

To validate the binding of EZH2 on genomic regions of its identified target genes, chromatin immunoprecipitation (CHIP) assay was performed with anti-EZH2 antibody as previously described^61^. Briefly, MCF-7 and MDA-MB-231 cells were grown for 90 percent confluence. DNA and proteins in the cells were cross-linked using 1% (v/v) formaldehyde and then sonicated in lysis buffer to obtain 200 bp–500 bp of DNA fragments. The lysate was equally divided for EZH2 immunoprecipitation and negative control IgG after keeping lysate for input control. Reverse-crosslinking and elution of DNA was followed by immunoprecipitation with antibodies. In the CHIP-seq data analyzed, binding sites for EZH2 was found at four downstream sites of PMEPA1, five sites of POMT2 and seven sites for CoL5A1. Two upstream sites each of SUMF1 & VGLL4 and two in-gene sites of GPNMB was found to be occupied by EZH2 in the published dataset. To validate the *in-vivo* binding of EZH2 on its identified target genes, CHIP-qPCR was performed with primers specific to the binding sites (Table S5). The fold enrichment was determined using the formula: Fold enrichment = 2(ΔCT of input- ΔCT of Immunoprecipitated DNA)

### Metabolic gEne RApid Visualizer expression database analysis and Correlation study

Publically available website MERAV (http://merav.wi.mit.edu/) which is designed to analyze human gene expression across 4,454 arrays and where all the arrays are normalized together to generate a database^62^, was analyzed for the correlation studies. Using the partial matrix available in the software, correlation coefficient values were computed. The breast cancer dataset includes more than 50 different established and patient derived breast cancer cell lines giving 198 data points; 332 primary breast tumors of different grades & histology types giving 332 data points; 23 expression data points from 23 non-cancerous breast cells and 142 data points from 142 normal breast tissues. Each relative expression value was used as single data point.

### Cell Culture

Human breast cancerous cell lines, (MCF-7, MDA-MB-231, T47D and MDA-MB-453) were obtained from National Repository of Animal Cell Culture, NCCS Pune (Maharashtra, India) and were independently authenticated by STR DNA fingerprinting at Institute of Life Sciences, Bhubaneswar, India. MCF-7 was maintained in Dulbecco’s Modified Eagle’s Medium (DMEM) and MDA-MB-231, T47D & MDA-MB-453 were maintained in Roswell Park Memorial Institute 1640 medium (RPMI) containing 10% fetal bovine serum supplemented with penicillin-streptomycin at 37 °C, 5% CO_2_ and 95% humidity. MCF10A, a kind gift from Dr. Annapoorni Rangaranjan (IISC, Bangalore, India) was maintained in DMEM F12 containing horse serum (5%) supplemented with hydrocortisone (0.5 mg/ml), EGF (20 ng/ml), insulin(10 µg/ml), cholera toxin (100 ng/ml) and penicillin-streptomycin at 37 °C, 5% CO_2_ and 95% humidity. Three technical as well as two biological repeat of the experiment was performed to get 6 data point for each cell line for correlation studies.

### Human breast cancer specimens and Immunohistochemistry

To investigate grade dependent EZH2 expression, fifteen histologically confirmed breast carcinoma samples were used to prepare tissue microarray (Table S1A) as previously described^63^. Further, to investigate the correlation of EZH2 expression with its target genes in real patient samples another 15 breast invasive ductal carcinoma cases along with their adjacent normal tissues were used for RNA extraction (Table S1B). In addition, tissues slides were obtained from US based company Origene. These slides contained tissue sections from tumors collected from appropriately matched smoking and never-smoked female breast cancer patients^32^. Immunohistochemistry in tissue array slides and slides from Origene was performed as described previously^64^. Slides were incubated with primary antibodies EZH2 (1:100) and SUMF1 (1:50) overnight at 4 °C and then subjected to incubation with anti-mouse/rabbit IgG secondary antibody for 1 hour. Diaminobenzidine was used to detect the immunoreactivity. Slides were subsequently counter stained with haematoxylin and after further processing mounting was done. Stained slides were observed under light microscope (Leica DM500) and images were captured at 4X and/or 40X magnification. Pathologist scored all the stained slides as described previously^32^.

### siRNA knockdown

The sequence of the EZH2si duplexes used in the study is provided in Table S3. MCF-7 and MDA-MB-231 cells were transfected with EZH2si using Lipofectamine 3000 (Invitrogen, US) siRNA transfection reagent (Invitrogen, US) following manufacturer’s protocol.

### Semi-quantitative and quantitative real-time PCR

Total RNA was extracted from cells using Trizol as previously described^65^. 1 µg of RNA was used to synthesize cDNA using kit (Invitrogen) as per the manufacturer’s instructions. qRT-PCR was performed on Roche platform using SYBR Green. Cycle threshold values were used to calculate the fold change in the gene expression after normalizing the values with those of Glyceraldehyde 3-phosphate dehydrogenase (GAPDH). Sequence of target specific real time PCR primers are shown in Table S4.

### Western Blot assay

Cells were lysed in RIPA lysis buffer containing 20 mM Tris-HCl (pH 7.5) 150 mM NaCl, 1 mM Na_2_EDTA, 1 mM EGTA, 1% triton X, 1% sodium deoxycholate, 2.5 mM sodium pyrophosphate, 1 mM β-glycerophosphate, 1 mM Na_3_VO4 and 1 µg/ml protease inhibitor. 50 µg of lysates was suspended in SDS-PAGE sample buffer containing 200 mM Tris-Cl (pH 6.8), 400 mM DTT, 8% SDS, 0.4% bromophenol blue, 40% glycerol and 0.05% mercaptoethanol, boiled at 95 °C for 5 minutes and electrophoresed on 10% SDS-polyacrylamide gel. The proteins were transferred onto Polyvinylidene difluoride (PVDF) membrane (88518, 0.45 µm, Thermo scientific). After blocking the membrane in 5% skimmed milk (Himedia) in tris-buffered saline (TBS) and Polysorbate 20 (Tween 20) TBS-T, incubation was done with primary antibodies overnight. The membrane was then washed three times with TBS-T and incubated with anti-rabbit or anti-mouse horseradish peroxidase conjugated secondary antibody for one hour. After washing the blot was developed for specific proteins using western bright ECL-HRP for X-ray Film Kit (K-12045-D50) (Advansta) in Chemi-Doc (BioRad).

### Km plotter analyses

The manually curated Kaplan Meier Plotter database^66^ was used to plot survival curve for identified unexplored EZH2 target genes. The database includes gene expression data and relapse free and overall survival information from GEO, EGA and TCGA. For breast cancer, the database includes 3995 patients for relapse free survival analyses.

### Mutation study

To find the existing mutations of EZH2 targets we explored cBioPortal^67^, a web resource for cancer genomics. We studied breast cancer previous studies altogether performed in 9051 patient samples such as Breast Cancer (MSK, Cancer Cell 2018); Breast Fibroepithelial Tumors (Duke-NUS, Nat Genet 2015); Breast Cancer (METAFABRIC, Nature 2012 & Nature Commun 2016); Breast Invasive Carcinoma (British Columbia, Nature 2012); Breast Invasive Carcinoma (Broad, Nature 2012); Breast Invasive Carcinoma (Sanger, Nature 2012); Breast Invasive Carcinoma (TCGA, Cell 2015); Breast Invasive Carcinoma (TCGA, Nature 2012); Breast Invasive Carcinoma (TCGA, PanCancer Atlas); Breast Invasive Carcinoma (TCGA, Provisional); Breast Cancer patient xenografts (British Columbia, Nature 2014); Mutational profiles of metastatic breast cancer; The Metastatic Breast Cancer Project (Provisional, April 2018) and Adenoid Cystic Carcinoma of the Breast (MSKCC, J Pathol. 2015).

### Statistical analyses

Throughout the current study, two tailed paired Student t-test and One-way ANOVA was performed to test the statistical difference between the experimental groups using the software GraphPad Prism v5.01. In addition, correlation coefficient (r) between EZH2 and its targets genes was calculated with the raw matrix data provided in MERAV database using GraphPad Prism v5.01.

## Supplementary information

Supplementary Data

## References

[1] Simon JA, Lange CA (2008). Roles of the EZH2 histone methyltransferase in cancer epigenetics. Mutation research.

[2] Volkel P, Dupret B, Le Bourhis X, Angrand PO (2015). Diverse involvement of EZH2 in cancer epigenetics. American journal of translational research.

[3] Dalman MR, Deeter A, Nimishakavi G, Duan ZH (2012). Fold change and p-value cutoffs significantly alter microarray interpretations. BMC bioinformatics.

[4] Tan J (2007). Pharmacologic disruption of Polycomb-repressive complex 2-mediated gene repression selectively induces apoptosis in cancer cells. Genes & development.

[5] Richly H, Aloia L, Di Croce L (2011). Roles of the Polycomb group proteins in stem cells and cancer. Cell death & disease.

[6] Jene-Sanz A (2013). Expression of polycomb targets predicts breast cancer prognosis. Molecular and cellular biology.

[7] Hock H (2012). A complex Polycomb issue: the two faces of EZH2 in cancer. Genes & development.

[8] Gong Y (2011). Polycomb group protein EZH2 is frequently expressed in inflammatory breast cancer and is predictive of worse clinical outcome. Cancer.

[9] Wassef M (2015). Impaired PRC2 activity promotes transcriptional instability and favors breast tumorigenesis. Genes & development.

[10] Yamaguchi H, Hung MC (2014). Regulation and Role of EZH2 in Cancer. Cancer research and treatment: official journal of Korean Cancer Association.

[11] Varambally S (2002). The polycomb group protein EZH2 is involved in progression of prostate cancer. Nature.

[12] Kim KH, Roberts CW (2016). Targeting EZH2 in cancer. Nature medicine.

[13] Han LC, Chen Y (2015). Targeting EZH2 for cancer therapy: progress and perspective. Current protein & peptide science.

[14] McCabe MT (2012). EZH2 inhibition as a therapeutic strategy for lymphoma with EZH2-activating mutations. Nature.

[15] Tiffen JC (2015). Targeting activating mutations of EZH2 leads to potent cell growth inhibition in human melanoma by derepression of tumor suppressor genes. Oncotarget.

[16] Nasveschuk CG (2014). Discovery and Optimization of Tetramethylpiperidinyl Benzamides as Inhibitors of EZH2. ACS medicinal chemistry letters.

[17] Stazi G, Zwergel C, Mai A, Valente S (2017). EZH2 inhibitors: a patent review (2014–2016). Expert opinion on therapeutic patents.

[18] Qi W (2012). Selective inhibition of Ezh2 by a small molecule inhibitor blocks tumor cells proliferation. Proceedings of the National Academy of Sciences of the United States of America.

[19] Deb G, Singh AK, Gupta S (2014). EZH2: not EZHY (easy) to deal. Molecular cancer research: MCR.

[20] Sorlie T (2003). Repeated observation of breast tumor subtypes in independent gene expression data sets. Proceedings of the National Academy of Sciences of the United States of America.

[21] Kumar R, Sharma A, Tiwari RK (2012). Application of microarray in breast cancer: An overview. Journal of pharmacy & bioallied sciences.

[22] Lehmann BD (2015). Evaluation of public cancer datasets and signatures identifies TP53 mutant signatures with robust prognostic and predictive value. BMC cancer.

[23] Korkola JE (2007). Identification of a robust gene signature that predicts breast cancer outcome in independent data sets. BMC cancer.

[24] Bertucci F (2003). Breast cancer revisited using DNA array-based gene expression profiling. International journal of cancer.

[25] Song X (2016). Selective inhibition of EZH2 by ZLD1039 blocks H3K27 methylation and leads to potent anti-tumor activity in breast cancer. Scientific reports.

[26] Gong C (2015). FOXA1 repression is associated with loss of BRCA1 and increased promoter methylation and chromatin silencing in breast cancer. Oncogene.

[27] Gu Y, Zhang J, Guan H (2017). Expression of EZH2 in endometrial carcinoma and its effects on proliferation and invasion of endometrial carcinoma cells. Oncology letters.

[28] Holm K (2012). Global H3K27 trimethylation and EZH2 abundance in breast tumor subtypes. Molecular oncology.

[29] Sheikh MS, Garcia M, Pujol P, Fontana JA, Rochefort H (1994). Why are estrogen-receptor-negative breast cancers more aggressive than the estrogen-receptor-positive breast cancers?. Invasion & metastasis.

[30] Mukaka MM (2012). Statistics corner: A guide to appropriate use of correlation coefficient in medical research. Malawi medical journal: the journal of Medical Association of Malawi.

[31] Dierks T (2005). Molecular basis for multiple sulfatase deficiency and mechanism for formylglycine generation of the human formylglycine-generating enzyme. Cell.

[32] Kumari K (2018). Nicotine associated breast cancer in smokers is mediated through high level of EZH2 expression which can be reversed by methyltransferase inhibitor DZNepA. Cell death & disease.

[33] McCarthy DJ, Smyth GK (2009). Testing significance relative to a fold-change threshold is a TREAT. Bioinformatics.

[34] Domcke S, Sinha R, Levine DA, Sander C, Schultz N (2013). Evaluating cell lines as tumour models by comparison of genomic profiles. Nature communications.

[35] Sandberg R, Ernberg I (2005). The molecular portrait of *in vitro* growth by meta-analysis of gene-expression profiles. Genome biology.

[36] Ross DT (2000). Systematic variation in gene expression patterns in human cancer cell lines. Nature genetics.

[37] Maric G, Rose AA, Annis MG, Siegel PM (2013). Glycoprotein non-metastatic b (GPNMB): A metastatic mediator and emerging therapeutic target in cancer. OncoTargets and therapy.

[38] Rose AA (2010). ADAM10 releases a soluble form of the GPNMB/Osteoactivin extracellular domain with angiogenic properties. PloS One.

[39] Rose AA (2010). Glycoprotein nonmetastatic B is an independent prognostic indicator of recurrence and a novel therapeutic target in breast cancer. Clinical cancer research: an official journal of the American Association for Cancer Research.

[40] Kuan CT (2006). Glycoprotein nonmetastatic melanoma protein B, a potential molecular therapeutic target in patients with glioblastoma multiforme. Clinical cancer research: an official journal of the American Association for Cancer Research.

[41] Qian X, Mills E, Torgov M, LaRochelle WJ, Jeffers M (2008). Pharmacologically enhanced expression of GPNMB increases the sensitivity of melanoma cells to the CR011-vcMMAE antibody-drug conjugate. Molecular oncology.

[42] Williams MD (2010). GPNMB expression in uveal melanoma: a potential for targeted therapy. Melanoma research.

[43] Cheng WY, Kandel JJ, Yamashiro DJ, Canoll P, Anastassiou D (2012). A multi-cancer mesenchymal transition gene expression signature is associated with prolonged time to recurrence in glioblastoma. PloS One.

[44] Cheon DJ (2014). A collagen-remodeling gene signature regulated by TGF-beta signaling is associated with metastasis and poor survival in serous ovarian cancer. Clinical cancer research: an official journal of the American Association for Cancer Research.

[45] Lommel M (2013). Protein O-mannosylation is crucial for E-cadherin-mediated cell adhesion. Proceedings of the National Academy of Sciences of the United States of America.

[46] Manya H (2004). Demonstration of mammalian protein O-mannosyltransferase activity: coexpression of POMT1 and POMT2 required for enzymatic activity. Proceedings of the National Academy of Sciences of the United States of America.

[47] Fournier PG (2015). The TGF-beta Signaling Regulator PMEPA1 Suppresses Prostate Cancer Metastases to Bone. Cancer cell.

[48] Nie Z (2016). Transforming growth factor-beta increases breast cancer stem cell population partially through upregulating PMEPA1 expression. Acta biochimica et biophysica Sinica.

[49] Ewald JA, Downs TM, Cetnar JP, Ricke WA (2013). Expression microarray meta-analysis identifies genes associated with Ras/MAPK and related pathways in progression of muscle-invasive bladder transition cell carcinoma. PloS One.

[50] Mann KM (2012). Sleeping Beauty mutagenesis reveals cooperating mutations and pathways in pancreatic adenocarcinoma. Proceedings of the National Academy of Sciences of the United States of America.

[51] Guo T (2013). A novel partner of Scalloped regulates Hippo signaling via antagonizing Scalloped-Yorkie activity. Cell research.

[52] Zhang W (2014). VGLL4 functions as a new tumor suppressor in lung cancer by negatively regulating the YAP-TEAD transcriptional complex. Cell research.

[53] Buono M, Visigalli I, Bergamasco R, Biffi A, Cosma MP (2010). Sulfatase modifying factor 1-mediated fibroblast growth factor signaling primes hematopoietic multilineage development. The Journal of experimental medicine.

[54] Sardiello M, Annunziata I, Roma G, Ballabio A (2005). Sulfatases and sulfatase modifying factors: an exclusive and promiscuous relationship. Human molecular genetics.

[55] Frankel LB (2014). A non-conserved miRNA regulates lysosomal function and impacts on a human lysosomal storage disorder. Nature communications.

[56] Schlotawa L (2011). SUMF1 mutations affecting stability and activity of formylglycine generating enzyme predict clinical outcome in multiple sulfatase deficiency. European journal of human genetics: EJHG.

[57] Mueller JW, Gilligan LC, Idkowiak J, Arlt W, Foster PA (2015). The Regulation of Steroid Action by Sulfation and Desulfation. Endocrine reviews.

[58] Lee ST (2011). Context-specific regulation of NF-kappaB target gene expression by EZH2 in breast cancers. Molecular cell.

[59] Kim JM (2015). Linker histone H1.2 establishes chromatin compaction and gene silencing through recognition of H3K27me3. Scientific reports.

[60] Li H (2012). ALDH1A1 is a novel EZH2 target gene in epithelial ovarian cancer identified by genome-wide approaches. Cancer prevention research.

[61] Carey MF, Peterson CL, Smale ST (2009). Chromatin immunoprecipitation (ChIP). Cold Spring Harbor protocols.

[62] Shaul YD (2016). MERAV: a tool for comparing gene expression across human tissues and cell types. Nucleic acids research.

[63] Hidalgo A, Pina P, Guerrero G, Lazos M, Salcedo M (2003). A simple method for the construction of small format tissue arrays. Journal of clinical pathology.

[64] Watson AS, Soilleux EJ (2015). Detection of p62 on Paraffin Sections by Immunohistochemistry. Cold Spring Harbor protocols.

[65] Rio DC, Ares M, Hannon GJ, Nilsen TW (2010). Purification of RNA using TRIzol (TRI reagent). Cold Spring Harbor protocols.

[66] Gyorffy B (2010). An online survival analysis tool to rapidly assess the effect of 22,277 genes on breast cancer prognosis using microarray data of 1,809 patients. Breast cancer research and treatment.

[67] Gao J (2013). Integrative analysis of complex cancer genomics and clinical profiles using the cBioPortal. Science signaling.

