# Supplementary material for: Genome-wide expression analysis reveals six contravened targets of EZH2 associated with breast cancer patient survival
Source: Sci Rep. 2019 Feb 13;9:1974. doi: 10.1038/s41598-019-39122-4 (PMC6374476; doi:10.1038/s41598-019-39122-4)
Supplement: Supplementary file 1 — Supplementary Data [file 41598_2019_39122_MOESM1_ESM.pdf]

# Genome-wide expression analysis reveals six contravened targets of EZH2 associated with breast cancer patient survival

**Kanchan Kumari[1,2], Biswajit Das[3,4], Amit Adhya[5], Arabinda K Rath [6], Sandip K Mishra*[1]**

[1]Cancer Biology Laboratory, Department of Gene Function and Regulation, Institute of Life Sciences, Bhubaneswar, Odisha, India

[2]Utkal University, Bhubaneswar, Odisha, India

[3]Tumor Microenvironment and Animal Models Laboratory, Department of Translational Research, Institute of Life Sciences, Bhubaneswar, Odisha, India

[4]Manipal University, Manipal, Karnataka, India

[5]Department of Pathology, AIIMS, Bhubaneswar, Odisha, India

[6]Hemalata Hospitals and Research Centre, Chandrashekharpur, Bhubaneswar, Odisha, India

* Corresponding author

***Sandip K. Mishra,** Ph.D. Scientist-F Cancer Biology Laboratory, Department of Gene Function and Regulation, Institute of Life Sciences, Bhubaneswar, Odisha, India. Email id. sandipkmishra@hotmail.com. Mobile No. 09437180218

| S. N0. | **Histologic type** | **Grade** | **Metastatic status** |
|---|---|---|---|
| 1 | Invasive ductal carcinoma | 2 | lymph node metastatic |
| 2 | Papillary carcinoma | 1 | |
| 3 | Invasive ductal carcinoma | 2 | lymph node metastatic |
| 4 | Invasive ductal carcinoma | 3 | lymph node metastatic |
| 5 | Invasive ductal carcinoma | 3 | |
| 6 | Invasive ductal carcinoma | 2 | |
| 7 | Invasive ductal carcinoma | 2 | |
| 8 | Invasive ductal carcinoma | 1 | |
| 9 | NORMAL AREA | | |
| 10 | Invasive ductal carcinoma | 2 | |
| 11 | Invasive ductal carcinoma | 2 | lymph node metastatic |
| 12 | NORMAL AREA | | |
| 13 | NORMAL AREA | | |
| 14 | Invasive ductal carcinoma | 2 | lymph node metastatic |
| 15 | Invasive ductal carcinoma | 3 | lymph node metastatic |

**Table S1 A**. **Tissue microarray patient sample details.** Eleven Invasive ductal carcinoma patient samples along with one sample from papillary carcinoma patient were included in the the study. To compare the expression of EZH2 in cancerous as well as non-cancerous breast tissue, three normal breast tissues were taken into consideration.

| | | | Adjacent NORMAL | Tumor | |
|---|---|---|---|---|---|
| S. No. | Sample ID | Grade | | | |
| 1 | 1364-16 | 2 | | X | |
| 2 | 1388-16 | 3 | | | |
| 3 | 3731/16 | 3 | | | |
| 4 | B-1479/16 | 3 | | | No amp in PMEPA1 |
| 5 | B-1513-16 | 1 | | | No amp in PMEPA1 |
| 6 | H-394/16 | 1 | | | No amp in PMEPA1 |
| 7 | 400/16 | 1 | X | | |
| 8 | B-257-17 | 2 | X | X | |
| 9 | B-336-17 | 3 | | | |
| 10 | B-399-17 | 3 | X | | |
| 11 | 482-17 | 1 | | | |
| 12 | B-898-17 | 0 | | | |
| 13 | H-125/17 | 2 | | | |
| 14 | H-127/17 | 3 | | | |
| 15 | H-138/17 | 1 | | | |

**Table S1 B**. **Patient sample details used for Transcript analysis.** Fifteen Histologically similar breast cancer patient samples of different grades were analyzed for the mRNA expression of EZH2 and its targets to validate the relevance of the online dataset. 'X' denotes no expression shown. Three of the samples showed no PMEPA1 expression.

| **Antibody/Reagent/Kit** | **Catalog no.** | **Company** |
|---|---|---|
| α-tubulin antibody | T5168 | Sigma Aldrich, USA |
| anti-EZH2 antibody<br>anti-SUMF1 antibody | D2C9-5246<br>PAC879Hu01 | Cell Signaling Technology<br>Cloud Clone Corp. |
| anti-Rabbit IgG (whole molecule)–Peroxidase antibody produced in goat secondary antibody | A0545 | Sigma Aldrich, USA |
| anti-mouse HRP conjugated secondary antibody | A3682-1ML | Sigma Aldrich, USA |
| 4, 5-dimethylthiazol-2yl)-2, 5-diphenyl tetrazolium bromide (MTT) | 2102227.1 | MP Biomedical, USA |
| SYBER Green | RT-SY2X-03+NRWOU | Eurogentech |
| SuperScript® First-Strand Synthesis System for RT-PCR | 11904018 | Invitrogen, Carlsbad, CA |
| Dnase I Kit | AMPD1-1KT | Sigma Aldrich, USA |
| INTERFERin Polyplus-Transfection reagent | 409-10 | Polyplus-transfection® SA |
| PVDF membrane | 88518, 0.45µm, | Thermo scientific |
| Skimmed milk | RM1254-500G | Himedia |
| ECL-HRP for X-ray Film Kit | K-12045-D50 | |
| Trizol | T9424 | Sigma Aldrich, USA |

**Table S2. Table provides the details of reagents used in the study.** All reagents used in the study were obtained from authentic companies.

| **Pooled EZH2si Duplexes (Eurogentech)** |
|---|
| 5’-GGG-AAA-GUG-UAU-GAU-AAA-U55-3’ |
| 5’-AUU-UAU-CAU-ACA-CUU-UCC-C55-3’ |
| 5’-CAC-AAG-UCA-UCC-CAU-UAA-A55-3’ |
| 5’-UUU-AAU-GGG-AUG-ACU-UGU-G55-3’ |
| 5’-GGA-UGG-UAC-UUU-CAU-UGA-A55-3’ |
| 5’-UUC-AAU-GAA-AGU-ACC-AUC-C55-3’ |

**Table S3: siRNA Duplexes.** Specific siRNA sequences targeting EZH2 Provided by Eurogentec was used for efficient EZH2 knockdown.

| Sl. No. | qRT-Primer Name | Primer Sequence 5' to 3' |
|---|---|---|
| 1 | GPNMB RT F | GAACCTTCAGCCTTAACC |
| 2 | GPNMB RT R | CTACAATTGTGATGGTGGC |
| 3 | PMEPA1 RT F | TGGAGTTTGTTCAGATC |
| 4 | PMEPA1 RT R | TGGGATTCCGTTGC |
| 5 | COL5A1 RT F | CTGCTCCCATGCCT |
| 6 | COL5A1 RT R | CCCCTCCAAGTCATC |
| 7 | VGLL4 RT F | GCCCACACTGACCC |
| 8 | VGLL4 RT R | ACCCAGAGCTTTGGC |
| 9 | POMT2 RT F | TCCAGCATGTTGACAGGTATCCT |
| 10 | POMT2 RT R | CATAAGCCAGAGGGTGGAAGAG |
| 11 | SUMF1 RT F | CCATCCCTGCTGGAGTA |
| 12 | SUMF1 RT R | GGTCTTCACTTGCTCACTC |
| 13 | 5’Ex1Ezh2 | AGAAGGGACCAGTTTGTTGG |
| 14 | 3’Ex1Ezh2 | TTCATCAGCTCGTCTGAACC |
| 15 | GAPDH RT FW | AAGATCATCAGCAATGCCTC |
| 16 | GAPDH RT RV | CTCTTCCTCTTGTGCTCTTG |

**Table S4: Real Time primer Sequence.** Quantitative Real time assay using specific primers was performed to study the expression of EZH2 and its identified targets. Primers against GAPDH were taken for normalization.

| S. No. | Oligo Name | Oligo Sequence 5' to 3' |
|---|---|---|
| 1 | CoL5A1 site 1 (F) | TGTGAAGTGTTGTCTATCTGAG |
| 2 | CoL5A1 site 1 (R) | CTAATGACGAAGGTGGTTTG |
| 3 | CoL5A1 site 2 (F) | GGGCTCAAGTAATCTGTCTG |
| 4 | CoL5A1 site 2 (R) | ATGGTGAAACCCTGTCTGTA |
| 5 | CoL5A1 site 3 (F) | TATTTTTGTAGAGGTGGGGTCT |
| 6 | CoL5A1 site 3 (R) | GGTAGGAGGATTGCTTGAGT |
| 7 | CoL5A1 site 4 (F) | CTTCTATGGGCTGTGGACAA |
| 8 | CoL5A1 site 4 (R) | GCAGCCATATCTCTAGGACC |
| 9 | CoL5A1 site 5 (F) | TGAGGGAACTATGTGAGTGAG |
| 10 | CoL5A1 site 5 (R) | TGGGGAGGACTCTCGT |
| 11 | CoL5A1 site 6 (F) | TCAAGGAGAGGATGGATGT |
| 12 | CoL5A1 site 6 (R) | AGGCAGACAGATGGACAC |
| 13 | GPNMB site 2 (F) | CCATAGTCTGTTGAATACCC |
| 14 | GPNMB site 2 (R) | TCTTGTAGCATTTGTTCTGG |
| 15 | GPNMB site 1 (F) | TTCCTTTTTGTTGGAGTCAG |
| 16 | GPNMB site 1 (R) | ATAAGTCAGGTGTGGTGTTGC |
| 17 | PEMAP1 site 1 (F) | ATTGGGCTTTACCTTTCTCT |
| 18 | PEMAP1 site 1 (R) | GGTTCACATCACAGGACTCT |
| 19 | PEMAP1 site 2 (F) | TGTGGTCAGAGAGAGTGCTT |
| 20 | PEMAP1 site 2 (R) | TAATAGTGGGGGACTTCAATAC |
| 21 | PEMAP1 site 3 (F) | AATAGTGCCCAGGTTGAGAC |
| 22 | PEMAP1 site 3 (R) | AGGTAGAGAAGAGGGTCAGG |
| 23 | PEMAP1 site 4 (F) | GAAGTTGGTGTGGGTCATTT |
| 24 | PEMAP1 site 4 (R) | GCTGTGTGTCCTCCATTTAG |
| 25 | POMT2 site 1 (F) | GTTCTCTACCTCCTCCCAGT |
| 26 | POMT2  site 1 (R) | ACAATAAGCAGGCATGAGTT |
| 27 | POMT2 site 2 (F) | CTAGGCAACAGAGCAAGACT |
| 28 | POMT2  site 2 (R) | CGAGATCATACCAATGGACT |
| 29 | POMT2 site 3 (F) | GAGGGAAATTAGGCGATATT |
| 30 | POMT2  site 3 (R) | TACCCTTTTGTGTGTGTGTG |
| 31 | POMT2 site 4 (F) | AGCTAAGATCACGCTACTGC |
| 32 | POMT2  site 4 (R) | TAGGTTCATCCATGTTGTCA |
| 33 | POMT2 site 5 (F) | CAGCTACTAAGGAGGCTGAG |
| 34 | POMT2  site 5 (R) | GGAGAGATTGAGCATTTCTG |
| 35 | SUMF1 site 1 (F) | TCAGGCAGCAAGTTTCTACT |
| 36 | SUMF1 site 1 (F) | GCTCTTTTACCTCACCTCCT |
| 37 | SUMF1 site 2 (F) | ATAAATTCCAGGACCCAAAG |
| 38 | SUMF1 site 2 (F) | CAGGGATGTGTGACTAAGG |
| 39 | VGLL4 site 1 (F) | GGAAGAAGAGGAAAAAGAGG |
| 40 | VGLL4 site 1 (R) | CCTCCCCAATCTAGTTCTTT |
| 41 | VGLL4 site 2 (F) | TCGGAGTGAAGAGAAGAAAA |
| 42 | VGLL4 site 2 (R) | TCATACTTCTAAGGGGCAAA |

**Table S5: Primer sequences for CHIP-qPCR:** Primers specific to binding sites of EZH2 on its identified target genes

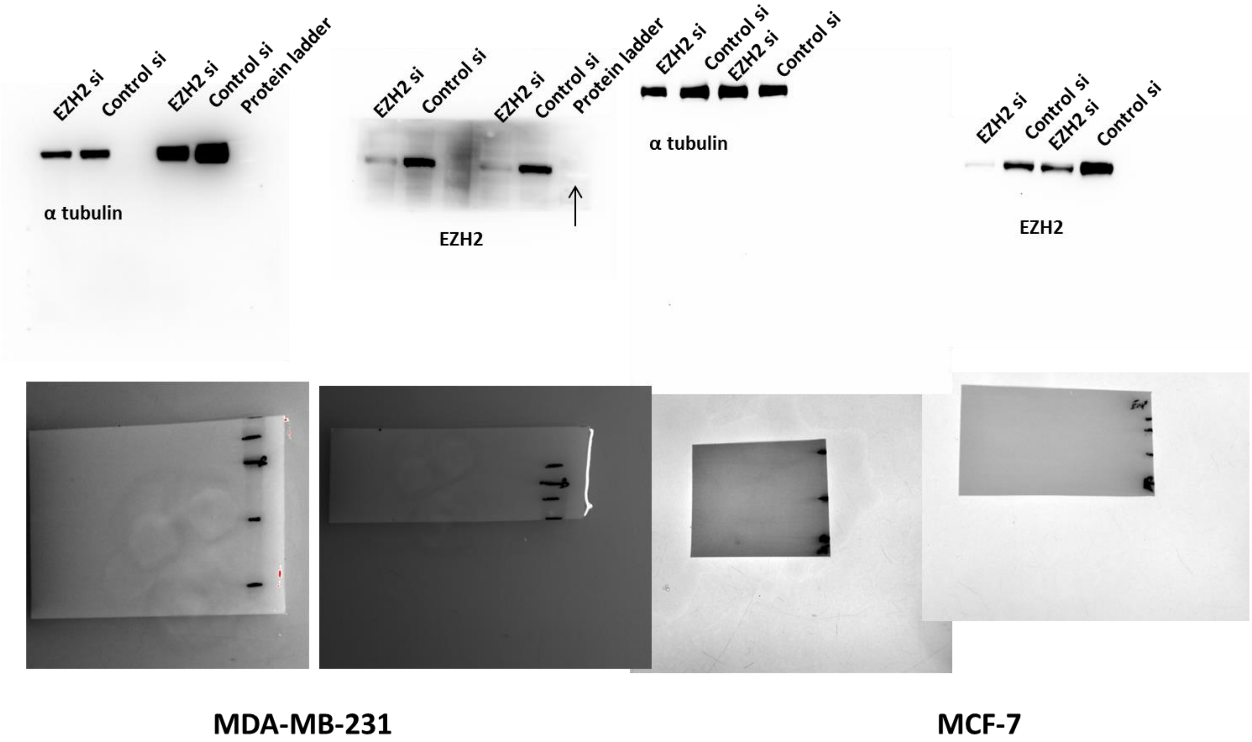


Blot images for western blot assay

**Figure S1**. Uncropped image of western blot done for knockdown studies displayed in Fig. 3A(i) and 3B(ii)

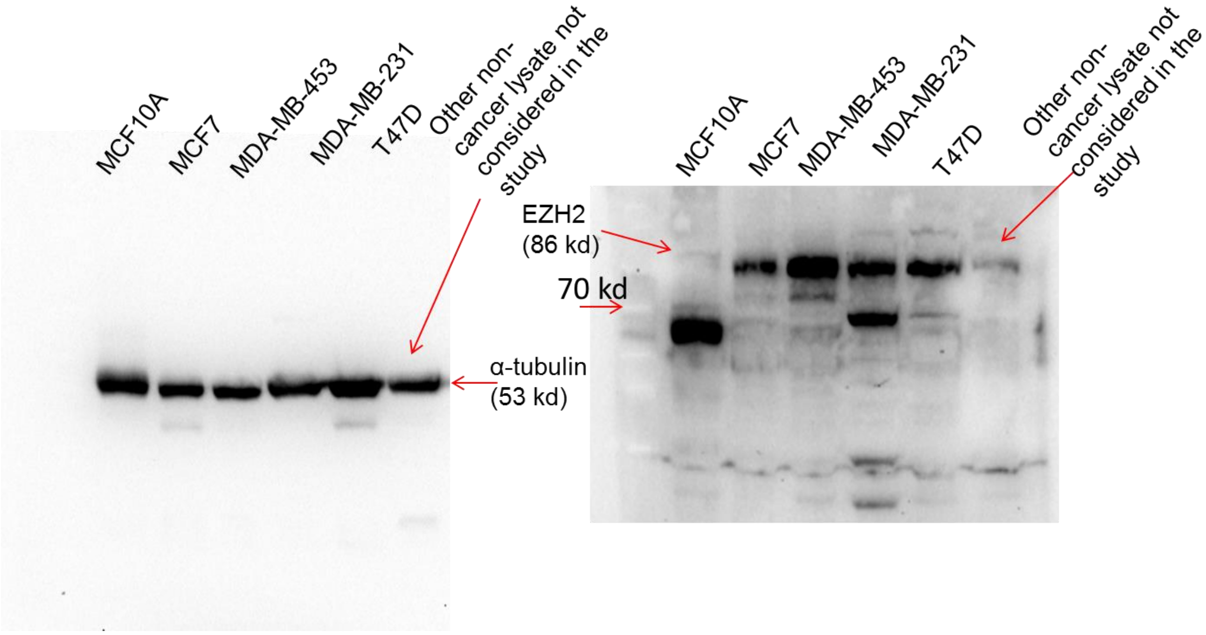


**Figure S2**. Uncropped image of immunoblot for EZH2 expression in different breast cancer cell lines displayed in Fig. 1B(i).

Quality of RNA used in the study

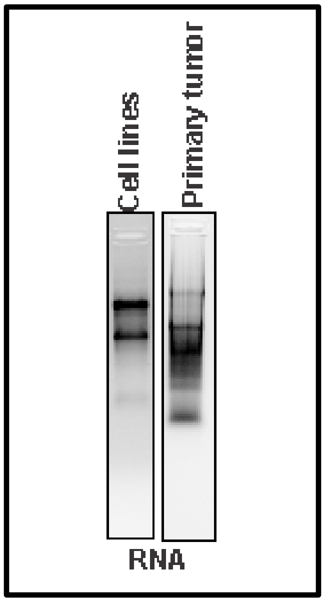


(i)

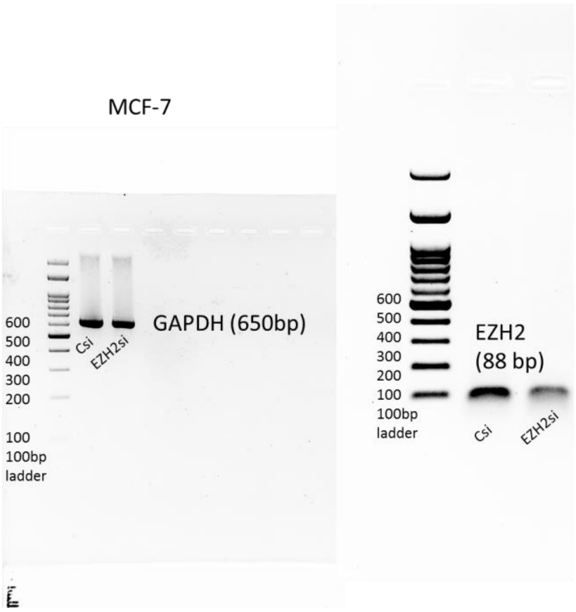


(ii)

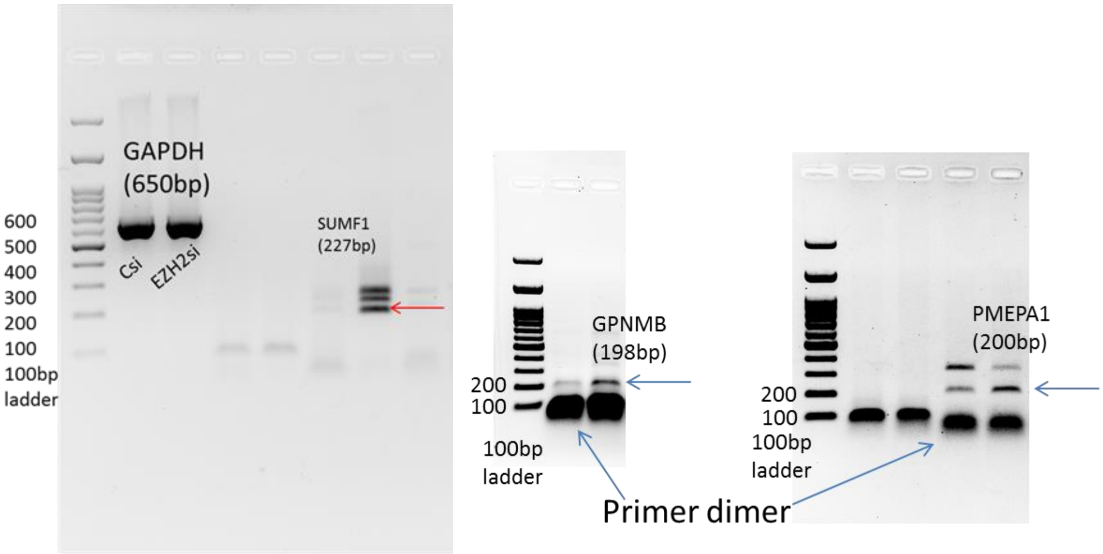

(iii)

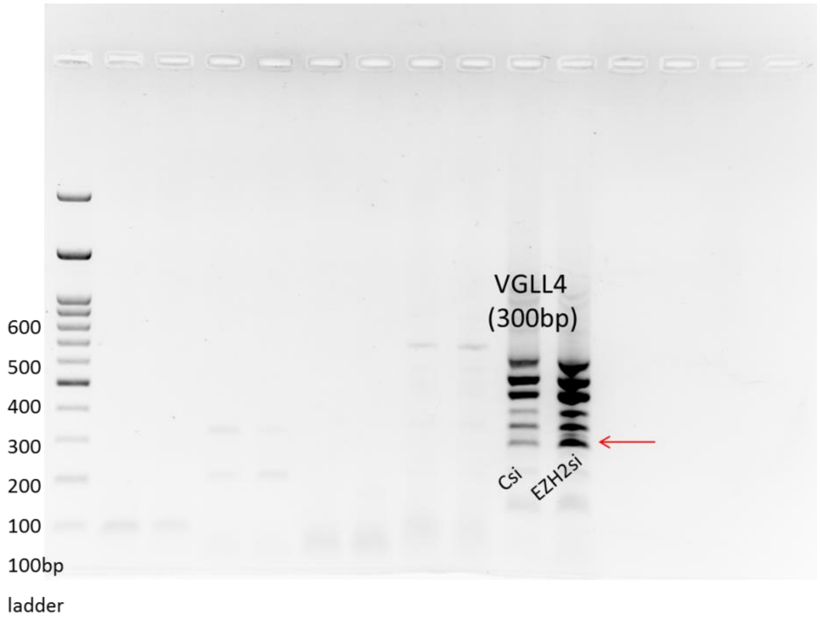


(iv)

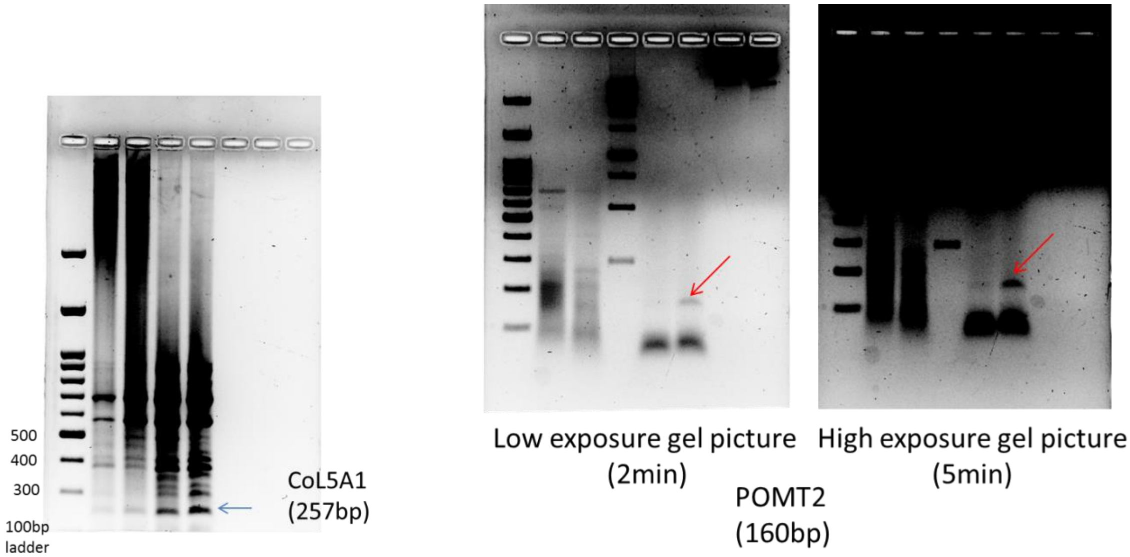


**Figure S3**. Uncropped images of agarose gel pictures for expression of (i) GAPDH, EZH2 (ii)SUMF1, GPNMB, PMEPA1 (iii) VGLL4 (iv) CoL5A1, POMT2 upon EZH2 silencing in MCF-7 breast cancer cells displayed in Figure 3E.

(i)

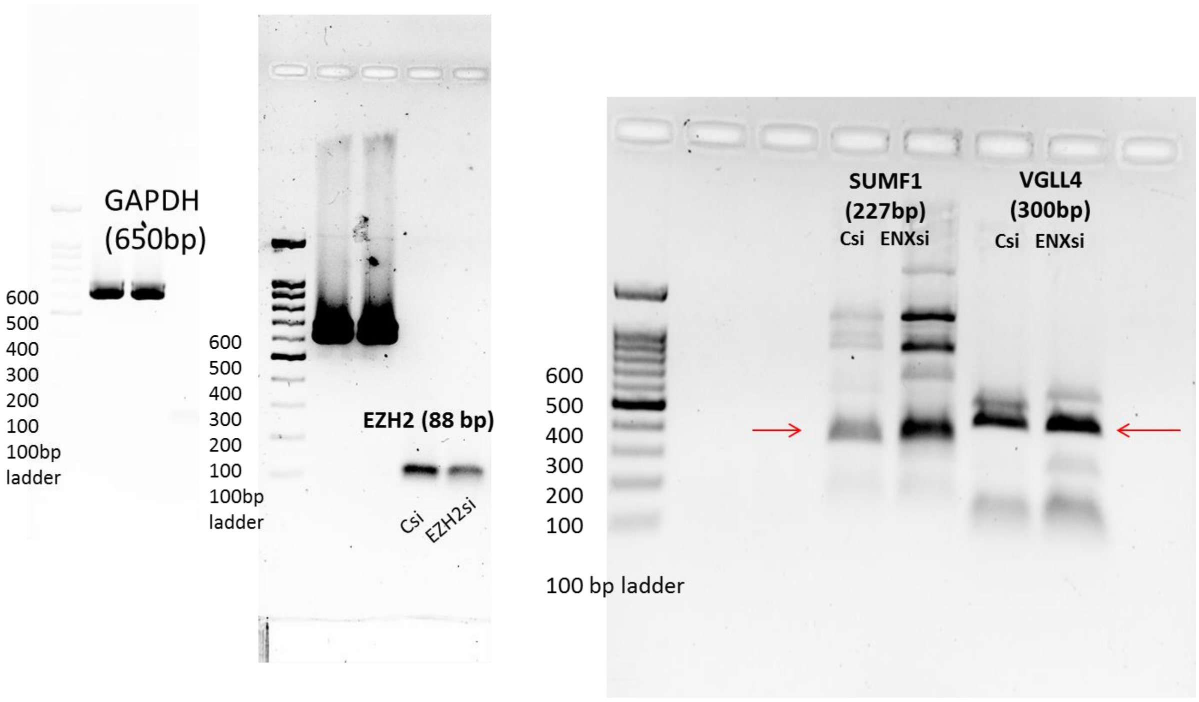
GAPDH
(650bp)
600
500
400
300
200
100
100bp
ladder
600
500
400
300
200
100
100bp
ladder
EZH2 (88 bp)
Csi
EZH2si
SUMF1
(227bp)
Csi ENXsi
VGLL4
(300bp)
Csi ENXsi
600
500
400
300
200
100
100 bp ladder

(ii)

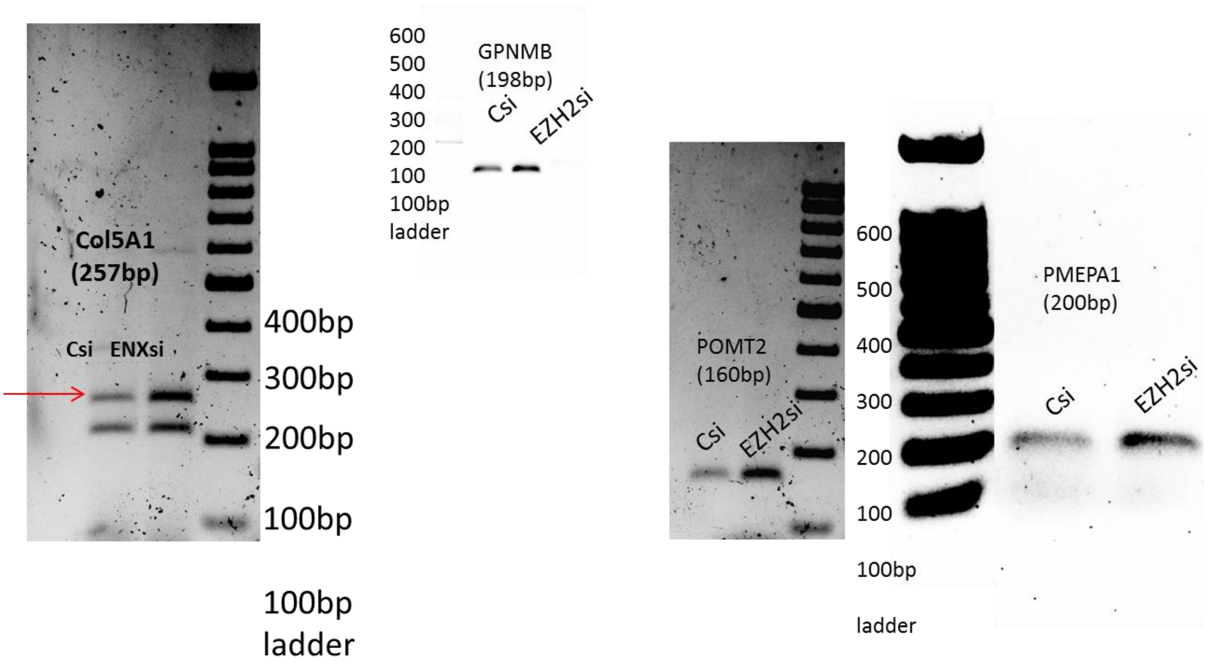
Col5A1
(257bp)
Csi ENXsi
400bp
300bp
200bp
100bp
100bp
ladder
600
500
400
300
200
100
100bp
ladder
GPNMB
(198bp)
Csi
EZH2si
POMT2
(160bp)
Csi
EZH2si
600
500
400
300
200
100
100bp
ladder
PMEPA1
(200bp)
Csi
EZH2si

**Figure S4**. Uncropped images of agarose gel pictures for expression of (i) GAPDH, EZH2, SUMF1, VGLL4 (ii) CoL5A1, GPNMB, POMT2, PMEPA1 upon EZH2 silencing in MDA-MB-231 breast cancer cells displayed in Figure 3F.

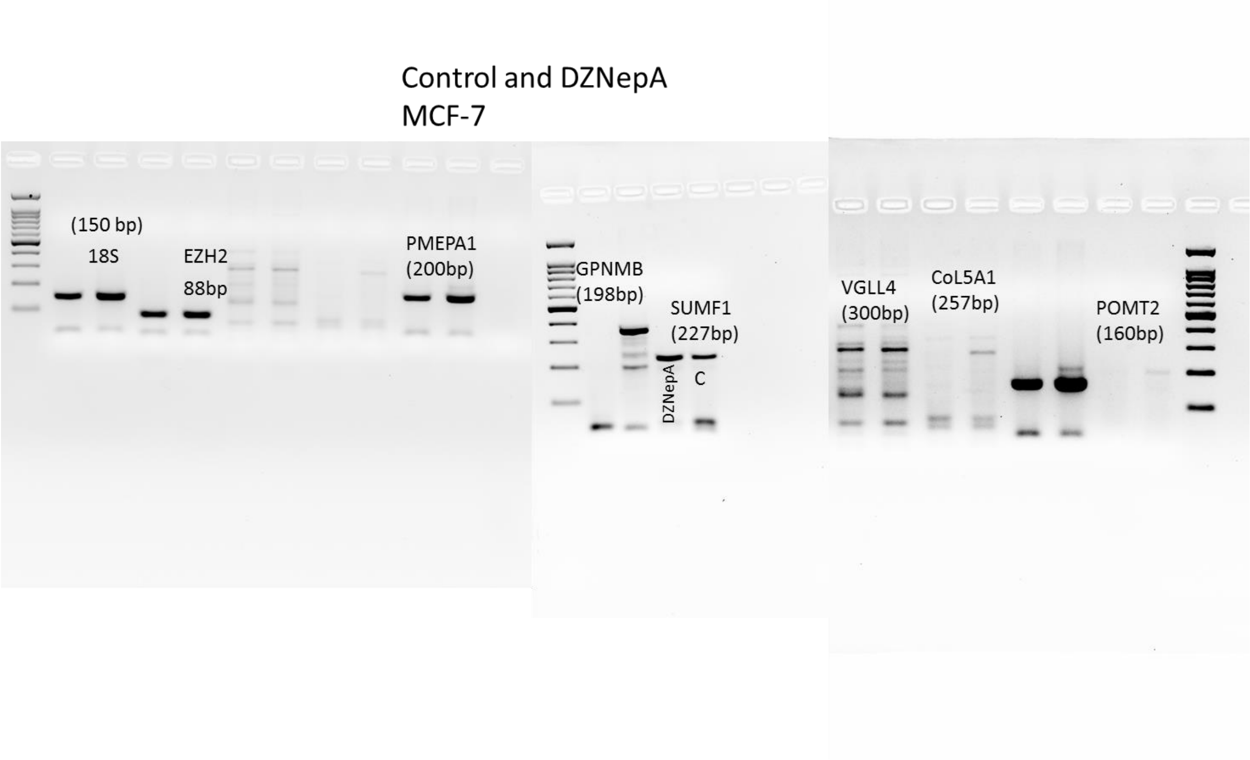


**Figure S5**. Uncropped images of agarose gel pictures for expression of 18S, EZH2, PMEPA1, GPNMB, SUMF1, VGLL4, CoL5A1 and POMT2 upon DZNepA treatment in MCF-7 breast cancer cells displayed in Figure 10A.

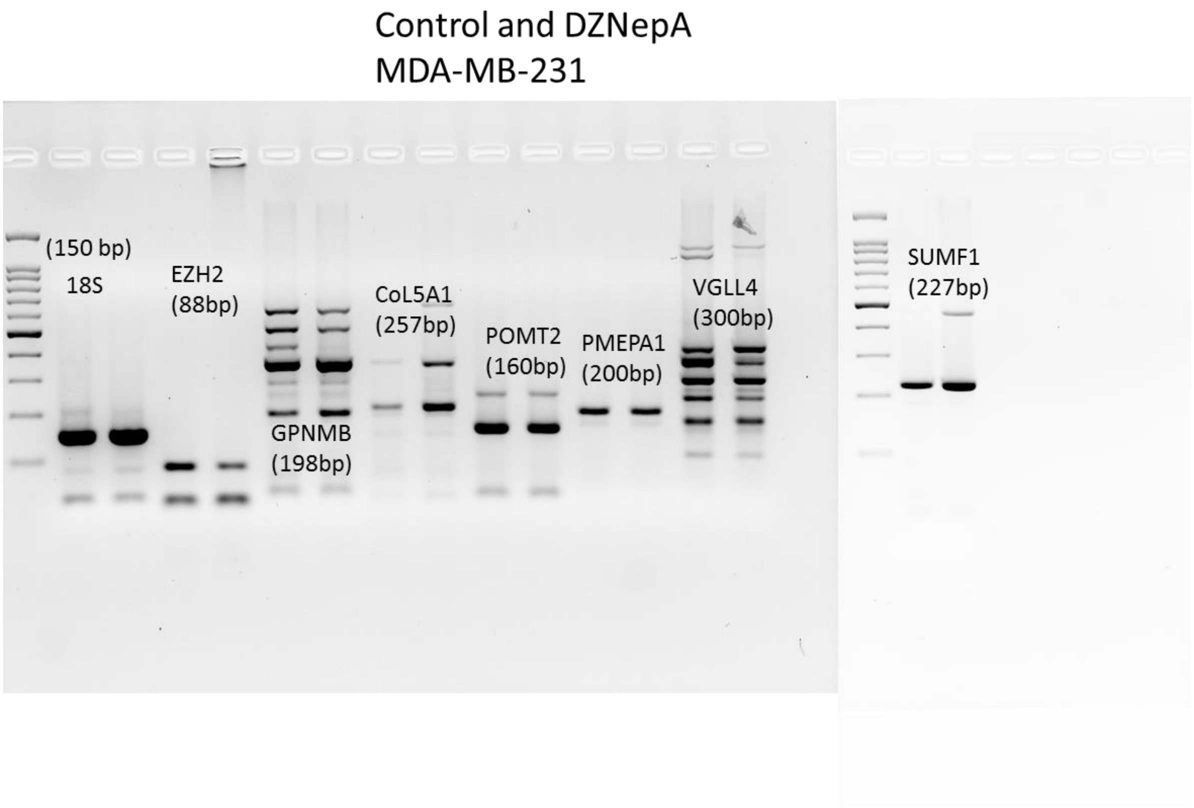


**Figure S6**. Uncropped images of agarose gel pictures for expression of 18S, EZH2, PMEPA1, GPNMB, SUMF1, VGLL4, CoL5A1 and POMT2 upon DZNepA treatment in MDA-MB-231 breast cancer cells displayed in Figure 10B.

Xenograft vehicle, Nicotine and Nicotine & DZNepA

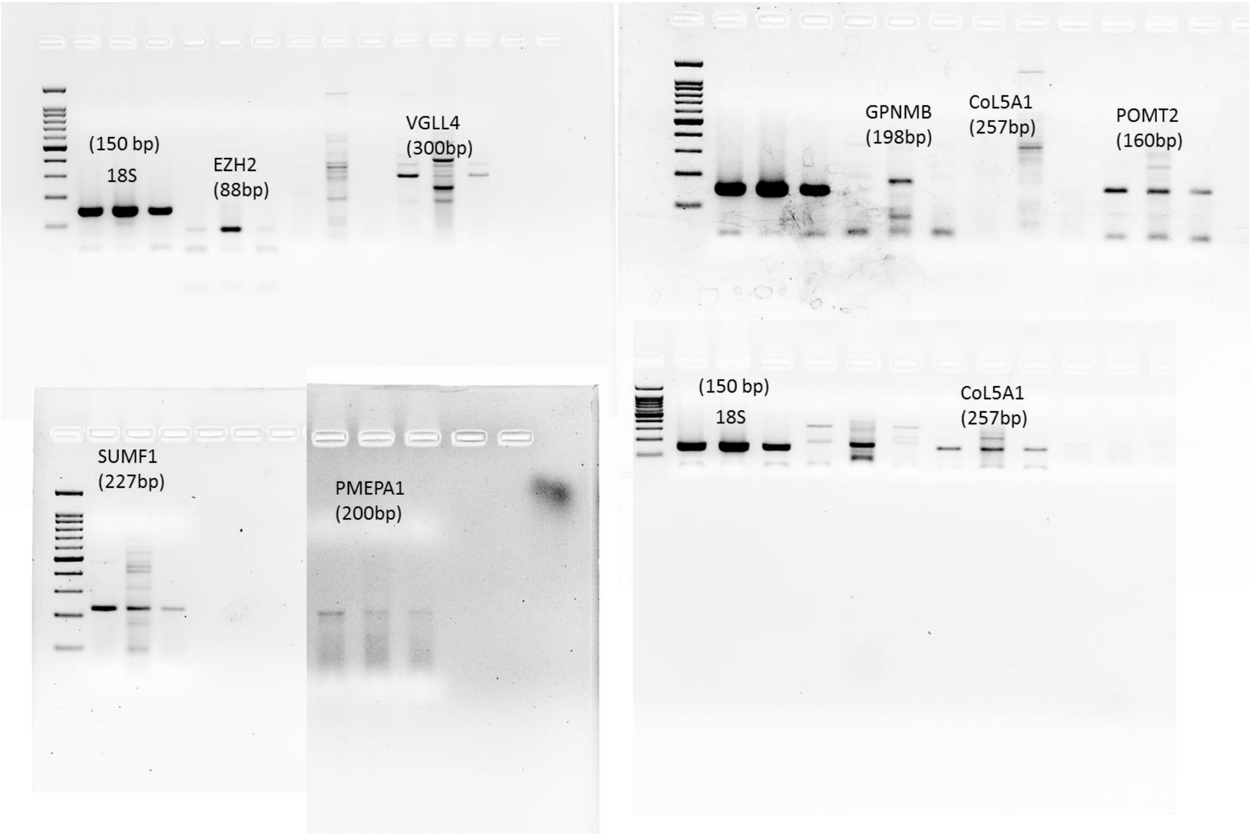


**Figure S7**. Uncropped images of agarose gel pictures for expression of 18S, EZH2, PMEPA1, GPNMB, SUMF1, VGLL4, CoL5A1 and POMT2 in xenograft extracted from vehicle, nicotine and nicotine& DZNepA treatment mice displayed in Figure 10C.
